# Dysregulation of Neuronal Cholesterol Homeostasis upon Exposure to HIV-1 Tat and Cocaine Revealed by RNA-Sequencing

**DOI:** 10.1038/s41598-018-34539-9

**Published:** 2018-11-02

**Authors:** Taha Mohseni Ahooyi, Masoud Shekarabi, Bahareh Torkzaban, T. Dianne Langford, Tricia H. Burdo, Jennifer Gordon, Prasun K. Datta, Shohreh Amini, Kamel Khalili

**Affiliations:** Department of Neuroscience, Center for Neurovirology, Comprehensive NeuroAIDS Center, Lewis Katz School of Medicine at Temple University, 3500N. Broad Street, Philadelphia, PA 19140 USA

## Abstract

HIV-1 Tat protein is released from HIV-1-infected cells and can enter non-permissive cells including neurons. Tat disrupts neuronal homeostasis and may contribute to the neuropathogenesis in people living with HIV (PLWH). The use of cocaine by PLWH exacerbates neuronal dysfunction. Here, we examined the mechanisms by which Tat and cocaine facilitate alterations in neuronal homeostatic processes. Bioinformatic interrogation of the results from RNA deep sequencing of rat hippocampal neurons exposed to Tat alone indicated the dysregulation of several genes involved in lipid and cholesterol metabolism. Following exposure to Tat and cocaine, the activation of cholesterol biosynthesis genes led to increased levels of free cholesterol and cholesteryl esters in rat neurons. Results from lipid metabolism arrays validated upregulation of several processes implicated in the biogenesis of β-amyloid and Alzheimer’s disease (AD), including sterol o-acyltransferase 1/acetyl-coenzyme A acyltransferase 1 (SOAT1/ACAT1), sortilin-related receptor L1 (SORL1) and low-density lipoprotein receptor-related protein 12 (LRP12). Further studies in Tat-treated primary neuronal cultures and brain tissues from HIV-1 transgenic mice as well as SIV-infected macaques confirmed elevated levels of SOAT1/ACAT 1 proteins. Our results offer novel insights into the molecular events involved in HIV and cocaine-mediated neuronal dysfunction that may also contribute to neuropathogenic events associated with the development of AD.

## Introduction

Antiretroviral therapy (ART) has significantly decreased the morbidity and mortality caused by HIV-1 infection, as well as progression toward AIDS^1^. Even though ART can efficiently block viral replication and restore CD4+ T-cell count, it has failed to eliminate the virus in all infected cells^2^. In turn, low levels of viral gene expression persist in people living with HIV (PLWH), even when plasma viral load is undetectable^3^. Thus, it is believed that some of the co-morbidities commonly observed in PLWH can be attributed to the presence of latent proviral DNA, called reservoirs, which express toxic viral proteins such as Tat that circulate and perturb homeostatic processes in surrounding cells and tissues^4^. In such cases, HIV-1 replication is inhibited by ART, while the proviral DNA integrated into the host genome continues to express low quantities of HIV proteins (e.g. Tat and Nef), subsequently leading to the emergence of diseases in the nervous, cardiovascular and endocrine systems, among others^5,6^. Furthermore, discontinuing ART can result in the reactivation of viral replication in most circumstances, suggesting a long-term risk for viral reactivation^7,8^.

HIV-associated neurologic dysfunction occurs in high rates among aging PLWH. These disorders are characterized by complications in the peripheral nervous system, as well as cognitive behavioral deficits across variety of domains including attention, learning and memory^9–12^. Gradual and persistent expression of the HIV-1 regulatory protein, Tat, has been recognized as a major cause in the emergence and development of neurocognitive disorders in PLWH taking ART. Although HIV-1 does not infect neurons, Tat released from infected cells in the brain, such as microglia, macrophage, and astrocytes, can enter into neurons and affects cellular functioning. Tat-induced neuronal injury and toxicity has been demonstrated in numerous *in vitro* and *in vivo* animal studies^13,14^. Tat has been shown to impair cell survival and communication pathways including bioenergetics^15^, calcium signaling^16,17^, apoptosis^18^ and neurotransmission^19,20^, however, the mechanisms by which Tat alters these processes to impair neuronal functioning remain poorly understood.

HIV-1-mediated changes in the transcriptome of HIV infected cells and tissues revealed widespread alterations in cellular pathways crucial for survival and functioning as well as processes that promote HIV-1 replication. Deep RNA sequencing of CD4+, CD8+ and CD14+ T-cells from PLWH demonstrated HIV-1-mediated changes in the transcriptome of these cells affecting metabolic, cell cycle and lipid profile pathways^21,22^. In addition, in HIV-1 infected macrophages, DNA and chromatin alterations have been reported^23^. Furthermore, earlier studies showed that treatment of peripheral blood lymphocytes from a healthy donor with HIV-1 Tat results in regulation of endogenous retroviruses including HEV-K^24^.

Transgenic rats harboring the HIV-1 genome served as a useful model to study the impact of HIV-1 on the central nervous system (CNS), and assessing neurobehavioral changes caused by the virus^25–27^. Using deep sequencing analysis of RNA transcripts, this model has been employed to identify altered patterns of gene expression in various regions of the brain^28,29^. Similarly, strategies were employed to evaluate impairments in expression of a variety of transcripts associated with working memory and cognition^30^. However, whether Tat alters the expression of specific processes impairing neuronal functioning that potentially contribute to molecular events associated with neurocognitive impairments, remains unknown.

The use of cocaine increases the risk for becoming infected by HIV and many studies have shown that together, HIV and cocaine exacerbate neurocognitive impairment^31^. Moreover, numerous *in vitro* and *in vivo* studies have reported that in combination, Tat and cocaine disrupt signaling cascades in the CNS and that these changes significantly affect functioning of uninfected bystander cells^32–34^. In fact, cocaine promotes viral replication in infected astrocytes^15^. Here, the interplay between Tat and cocaine in the context of disruption of neuronal homeostasis was addressed.

Lipid and cholesterol homeostasis are tightly controlled in brain in general and neurons in particular^35,36^. Impairment of this homeostasis has been widely linked to neurodegeneration and neurological disorders such as Alzheimer’s disease, Huntington’s disease and Parkinson’s disease, among others^37–39^. Through activation of Nitric Oxide (NO) synthesis, HIV has been associated with mitochondrial cholesterol accumulation in neurons^40^. HIV was also implied in the accumulation of cholesterol and ceramide in the infected individuals CNS as an indicator of HIV-associated neurocognitive disorders^41^. Antiretroviral therapy (ART) has been shown to increase HDL^42^ while reducing low density lipoprotein (LDL) cholesterol^43^. ART was also reported to improve cholesterol efflux capacity in individual infected with HIV^44^.

In this research, we utilized embryonic rat hippocampal neuron cultures to initially examine variations in the neuronal transcriptome following exposure to Tat and cocaine. Using a statistical clustering approach, a number of significantly dysregulated pathways were identified, most notable were those involved in lipid metabolism and cholesterol synthesis, as well as several other pathways involved in cytokine signaling and inflammatory responses, G-protein coupled receptors and olfactory receptors. A qRT-PCR lipid metabolism array revealed the upregulation of several genes that are implicated in the biogenesis of β-amyloid and Alzheimer’s disease (AD), including sterol o-acyltransferase 1/acetyl-coenzyme A acyltransferase 1 (SOAT1/ACAT1), sortilin-related receptor, L1 (SORL1) and low-density lipoprotein receptor-related protein 12 (LPR12). Results from analyses of HIV-1 transgenic mice (Tg26) cortical tissue and Tat-treated rat primary neuronal culture clearly demonstrated an increase in SOAT1/ACAT1 mRNA and protein levels. Furthermore, qRT-PCR array, western blotting and immunofluorescence imaging analyses validated these observations in Tat-treated rat neurons, Tg26 mice and SIV-infected macaque brain tissues. In a coordinated manner, the activation of cholesterol biosynthesis genes led to increased levels of free cholesterol and cholesteryl esters (CE) in rat neurons following exposure to Tat and cocaine. The current observations provide novel insights into the mechanisms involved in the manifestation of HIV-mediated neurocognitive disorders and suggest that similar pathological mechanisms may occur in the neuropathogenesis of AD.

## Results

### Global transcriptome analysis of hippocampal neurons in response to Tat and Cocaine

**Figure 1:**
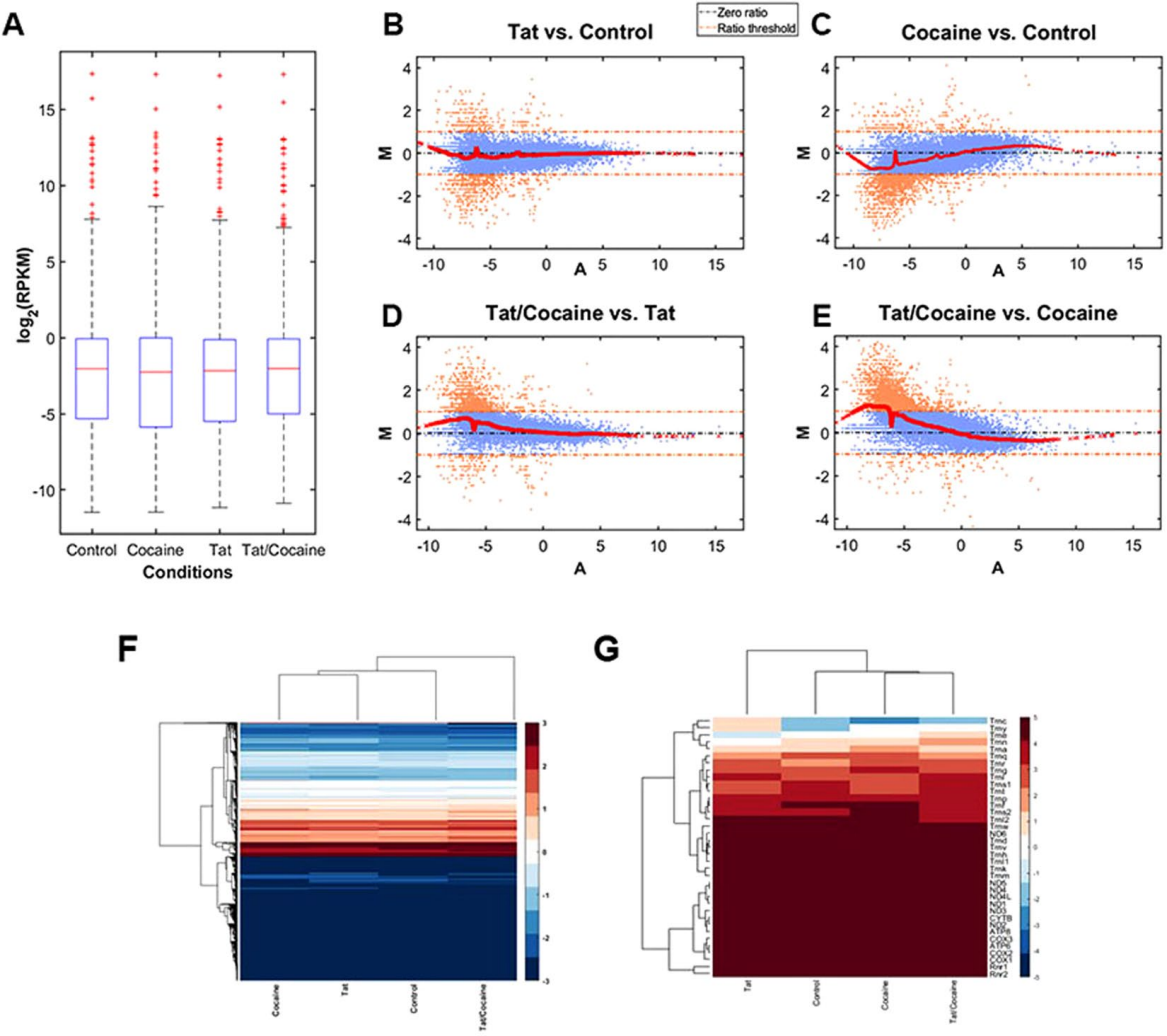
Expression profiling of the cellular mRNAs for control, cocaine, Tat and Tat/cocaine. (**A**) Box plot showing the statistical features of the expression profiles under different experimental conditions. MA plots of Tat vs. control, (**B**) cocaine vs. control, (**C**). Tat/cocaine vs. Tat, and (**D**) Tat/cocaine vs. cocaine, (**E**) Hierarchical clustering of (**F**) Cellular mRNAs and (**G**) Mitochondrial mRNAs are illustrated. RPKM, Reads per Kilobase Million; MA, log ratio vs. mean average.

Primary cells derived from rat embryonic day 18 (E18) hippocampal tissue is highly enriched in neurons and has served as an *in vitro* model for studying many neuronal biological functions including formation of synaptic contacts and connectivity^45^. Here, we performed RNA-seq analysis of transcripts from rat hippocampal neurons after exposure to recombinant HIV-1 Tat protein and cocaine, either alone or in combination. The aligned RNA-seq data from neurons were transformed into the log_2_ of RPKM (Reads per Kilobase Million) unit and uni- and multivariate analysis were conducted (Fig. 1). The box plot of the distribution of log_2_ (RPKM) for control, cocaine, Tat and Tat/cocaine conditions shows mRNA levels at the minimum, maximum, median and first and third quantiles of the data (Fig. 1A). The checkpoints of distribution for the experimental conditions show no statistically significant differences. The similarity of the log_2_ (RPKM) distributions were also be captured by their probability density functions estimated using the Kernel method (Fig. S1).

Detailed multivariate analysis was performed by comparative study of the pairwise log_2_(RPKM)s and the results are presented by *MA* (log ratio vs. mean average) plots of cocaine vs. control, Tat vs. control, Tat/cocaine vs. cocaine and Tat/cocaine vs. Tat (Fig. 1B–E). A greater than two-fold downregulation compared to two-fold upregulation of genes was observed in different conditions (Fig. 1B,C). These results suggest that separately, Tat and cocaine induce lower transcription rates or decrease mRNA stability. On the other hand, Tat/cocaine co-treatment led to a higher ratio of upregulated transcripts when compared to Tat or cocaine alone (Fig. 1D,E).

Next, we performed the hierarchical clustering of the cellular mRNA and mitochondrial mRNA expression levels after log_2_(RPKM) transformation, respectively (Fig. 1F,G). We then compared the experimental conditions using hierarchical clustering complemented with heat-map visualization of the clustered gene groups and the co-expressed genes. This analysis provides a basis for the detection of clusters of genes that exhibit similar expression patterns under different conditions. As revealed by this analysis, cellular gene responses to Tat or cocaine are similar compared to control; whereas, Tat/cocaine co-treatment upregulates the expression of several specific families of genes (Fig. 1F and Table 1, gene names are not listed for presentation purposes). Moreover, mitochondrial gene responses to cocaine and Tat/cocaine conditions show higher correlations with one another suggesting that cocaine may be regulating mitochondrial gene expression profiles (Fig. 1G). In this context, the overall effects of Tat, cocaine, and Tat/cocaine were upregulation of mitochondrial gene expression, mostly affecting the tRNA family (Trny, Trne, Trnn, Trna, Trnq) that normally show low baseline expression levels. These effects may suggest changes to mitochondrial protein synthesis, but this requires further investigation. On the other hand, the highly expressed MT-COX and MT-ATP mRNAs remained unchanged across all treatments (Fig. 1G).

**Table 1:** Functional annotations of the cellular processes pathways most significantly affected by cocaine, Tat and Tat/cocaine as compared with control.

Cocaine	p-value	Tat	p-value	Tat/Cocaine	p-value
Extracellular Region	5.00e-41	Regulation of Synaptic Transmission, Cholinergic	0	Response to Ether	0
Multicellular Organismal Process	8.00e-18	Response to Lipopolysaccharide	0	Involved in Ventral Spinal Cord Interneuron Specification	0
Immune Response	7.00e-16	Transcription Factor Complex	0	Sequence-specific DNA Binding	0
Defense Response	2.00e-15	Negative Regulation of Glutamate Secretion	0	Stem Cell Fate Specification	3.00e-05
Sequence-specific DNA Binding	1.00e-14	Spinal Cord Association Neuron Differentiation	0	*Growth Factor Activity*	*0*
Positive Regulation of Immune System Process	1.00e-12	Neutrophil Chemotaxis	0	*Extracellular Region*	*7*.*00e-34*
Serine-type Endopeptidase Activity	3.00e-12	Extracellular Region	5.00e-23	*Extracellular Space*	*5*.*00e-14*
Developmental Process	1.00e-10	Extracellular Space	3.00e-13	*G-protein Coupled Receptor Protein Signaling Pathway*	*8*.*00e-14*
DNA Binding Transcription Factor Activity	2.00e-10	Sequence-specific DNA Binding	8.00e-11	*G-protein Coupled Receptor Activity*	*1*.*00e-12*
Regulation of Multicellular Organismal Process	7.00e-10	DNA Binding Transcription Factor Activity	7.00e-08	*Hormone Activity*	*1*.*00e-11*
Peptidase Inhibitor Activity	1.00e-09	Serine-type Endopeptidase Activity	1.00e-07	*Defense Response*	*3*.*00e-11*
Immune Effector Process	1.00e-07	Endopeptidase Inhibitor Activity	9.00e-07	*Peptidase Inhibitor Activity*	*1*.*00e-09*
Hormone Activity	1.00e-07	Regulation of The Force Of Heart Contraction	4.00e-05	*Detection of Chemical Stimulus*	*1*.*00e-09*
Eicosanoid Metabolic Process	2.00e-07	DNA Bending Activity	4.00e-05	*Response to Bacterium*	*1*.*00e-08*
Response to Organic Substance	2.00e-07	Chemokine Activity	9.00e-05	*Immune Response*	*5*.*00e-08*
Chemokine Activity	3.00e-07	Cellular Calcium Ion Homeostasis	9.00e-05	*Cytokine Activity*	*7*.*00e-08*
Monooxygenase Activity	7.00e-07	Positive Regulation of Natural Killer Cell Chemotaxis	9.00e-05	*Cytokine Receptor Binding*	*1*.*00e-07*
Heparin Binding	9.00e-07	*Positive Regulation of Podosome Assembly*	*0*	*Intrinsic to Membrane*	*3*.*00e-07*
Response to Endogenous Stimulus	9.00e-07	*Humoral Immune Response*	*0*	*External Side of Plasma Membrane*	*1*.*00e-06*
Protein Activation Cascade	4.00e-06	*Chronic Inflammatory Response to Antigenic Stimulus*	*0*	*Positive Regulation of Cytokine Biosynthetic Process*	*4*.*00e-06*
External Side of Plasma Membrane	8.00e-06	*Diencephalon Development*	*0*	*Regulation of Immunoglobulin Production*	*1*.*00e-05*
Stored Secretory Granule	2.00e-05	*G-protein Coupled Receptor Activity*	*1*.*00e-10*	*Negative Regulation of Endopeptidase Activity*	*2*.*00e-05*
Drug Metabolic Process	2.00e-05	*G-protein Coupled Receptor Protein Signaling Pathway*	*2*.*00e-10*	*Response to External Stimulus*	*7*.*00e-05*
Positive Regulation of Cellular Biosynthetic Process	3.00e-05	*Chemical Stimulus Involved in Sensory Perception*	*4*.*00e-10*	*Sugar Binding*	*8*.*00e-05*
*Integral to Membrane*	*2*.*00e-05*	*Defense Response to Bacterium*	*2*.*00e-07*	*Response to Lipopolysaccharide*	*8*.*00e-05*
*Neuron Fate Determination*	*1*.*00e-05*	*Hormone Activity*	*7*.*00e-07*	*Regulation of Chemokine Biosynthetic Process*	*8*.*00e-05*
*Olfactory Receptor Activity*	*1*.*00e-05*	*Neuron Fate Determination*	*3*.*00e-05*	*Cellular Response to Cytokine Stimulus*	*9*.*00e-05*
*Detection of Stimulus*	*3*.*00e-06*	*Cytokine Activity*	*7*.*00e-05*		
*Cholesterol Biosynthetic Process*	*6*.*00e-16*				

Pathways in plain bold and bold italics typeface are down- and upregulated, respectively.

### Functional annotations of highly modulated cellular pathways

The highly affected cellular processes in response to cocaine, Tat and Tat/cocaine combination are listed (Table 1). Notably, Tat and Tat/cocaine increased the mRNA of genes that express G-protein coupled receptors, a phenomenon which was absent in cocaine only treated neurons and control. Similarly, Tat and Tat/cocaine upregulated mRNAs for cytokine receptors including IL-3, IL-5 and IL-6, hence cellular response to cytokine stimulus (Fig. S2). Overall, Tat and Tat/cocaine upregulated the inflammatory pathways in neurons as represented by the cytokine activity and response to bacterial infections. This effect is mostly linked to the upregulation of TNF-α and interleukin family (Fig. S2). With cocaine alone, lower levels of mRNAs involved in the inflammation are observed, suggesting that Tat may be responsible for the upregulation. All three conditions promoted an increase in the rat chemical sense pathways including olfactory family genes. Hormone activity related mRNA levels were also increased in response to Tat and Tat/cocaine, while they were reduced by cocaine alone. Chemokine activity related mRNAs were downregulated by Tat and cocaine, while increased by Tat/cocaine co-treatments (Table 1). Overall, the results indicate a dominant effect of Tat on these pathways compared to cocaine, as Tat restores some of the cocaine inhibitory effects. In conclusion, Tat/cocaine promoted a stimulatory state compared to cocaine alone which showed inhibitory properties.

In addition, multiple regulatory pathways are modulated by Tat, cocaine and Tat/cocaine, compared to control. As indicated in Table S1, zinc ion binding-associated mRNAs are upregulated by Tat, cocaine and Tat/cocaine compared to the control. Thus, it is evident that Tat increases transcription and DNA-dependent pathways, and cocaine ameliorates this effect. Additionally, both cocaine and Tat led to significant increases in the RNA splicing and genes related to pre-mRNA 3′-splice binding sites. Also, cocaine upregulated spliceosome assembly related mRNAs. Cocaine, Tat and Tat/cocaine downregulated protein kinase signaling cascades (PKC in Tat and cocaine and PKA in Tat/cocaine), while cocaine increased mRNAs related to this pathway via the NFκB pathway (Table S1). We also observed that cocaine-induced upregulation of nerve growth factor receptor mRNA, while Tat caused induction of pathways related to CNS asymmetry in the neuronal development.

### Differentially expressed gene (DEG)-based identification of neuronal pathways affected by Tat and Cocaine

Two distinct approaches were employed to identify highly altered genes by Tat and cocaine. For example, in the first approach, for each experimental condition, a list of DEGs that were up- or downregulated more than two-fold compared to the control were constructed. The lists were fed into BiNGO^46^, a Cytoscape application, for functional annotation of the overrepresented pathways. Both Tat (Fig. S3A) and cocaine (Fig. S3C) downregulated mRNAs in pathways related to the immune response and development/differentiation processes. Cocaine downregulated the expression of mRNAs associated with fatty acid metabolic pathways (Fig. S3C). Interestingly, both Tat and cocaine increased mRNAs related to pathways involved in stimulus detection (Fig. S3B,D). Treatment with cocaine increased mRNAs associated with lipid metabolism where lipid biosynthetic processes, steroid metabolic processes and cholesterol biosynthesis are highly overrepresented (Fig. S3D).

In the second approach, the k-means clustering method was utilized to generate a list of genes that highly and coordinately responded to the various experimental conditions. We selected 20 clusters based on the number of genes and their expression profiles (Fig. S4). In particular, we were interested in profiles that significantly responded to the Tat and cocaine treatments. Functional annotation of the groups of genes clustered in either a similar or a highly Tat/cocaine-responsive manner and led to the identification of additional overrepresented pathways. The sterol and more specifically, cholesterol metabolic pathways, lie at the center of the positively responding genes to both Tat and Tat/cocaine. Taken together, the RNA-Seq data along with the pathway analysis signifies the impact of Tat and Tat/cocaine on induction of lipid pathways.

### Tat and cocaine significantly alter hippocampal neuronal lipid metabolism

**Figure 2:**
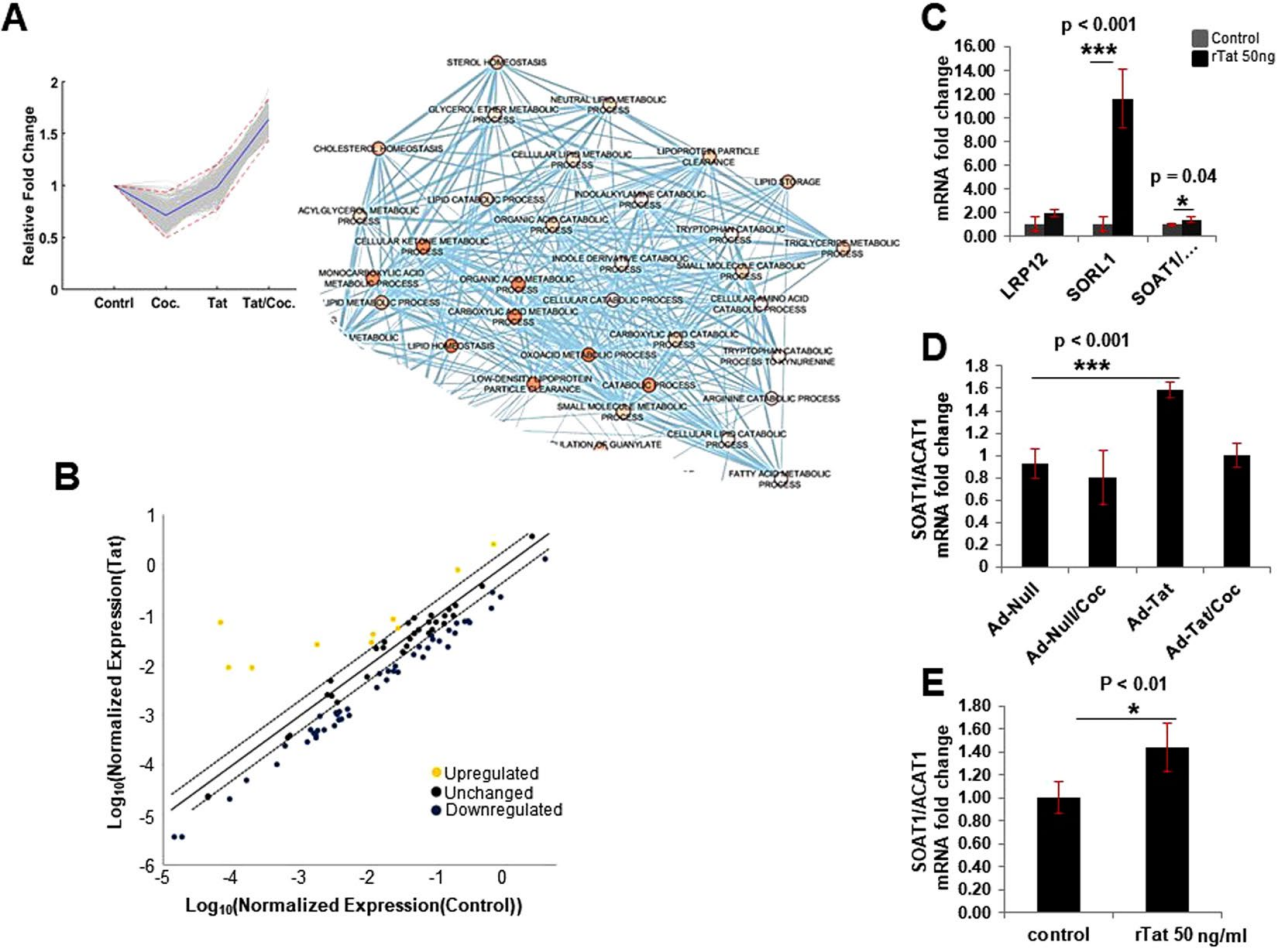
Tat and cocaine dysregulate cholesterol metabolism by altering key genes involved in APP trafficking (SORL1) and cholesterol esterification (SOAT1/ACAT1). (**A**) Functional annotation of k-means clustering identified a group of genes involved in the dysregulation of lipoprotein metabolism pathways. (**B**) Lipoprotein signaling and cholesterol metabolism pathway PCR array validated dysregulation of key genes in this pathway. (**C**) qRT-PCR confirmed the upregulation of SOAT1/ACAT1, SORL1 and LRP12 in HIV-1 Tat protein treated rat neurons. (**D**) Adeno-Tat/cocaine treatment of rat neurons showed that cocaine has an inhibitory effect on Tat-mediated overexpression of SOAT1/ACAT1. (**E**) Relative SOAT1/ACAT1 mRNA expression in rat primary neurons in the presence of Tat protein. The statistical analyses were conducted via t-test with n = 3.

**Figure 3:**
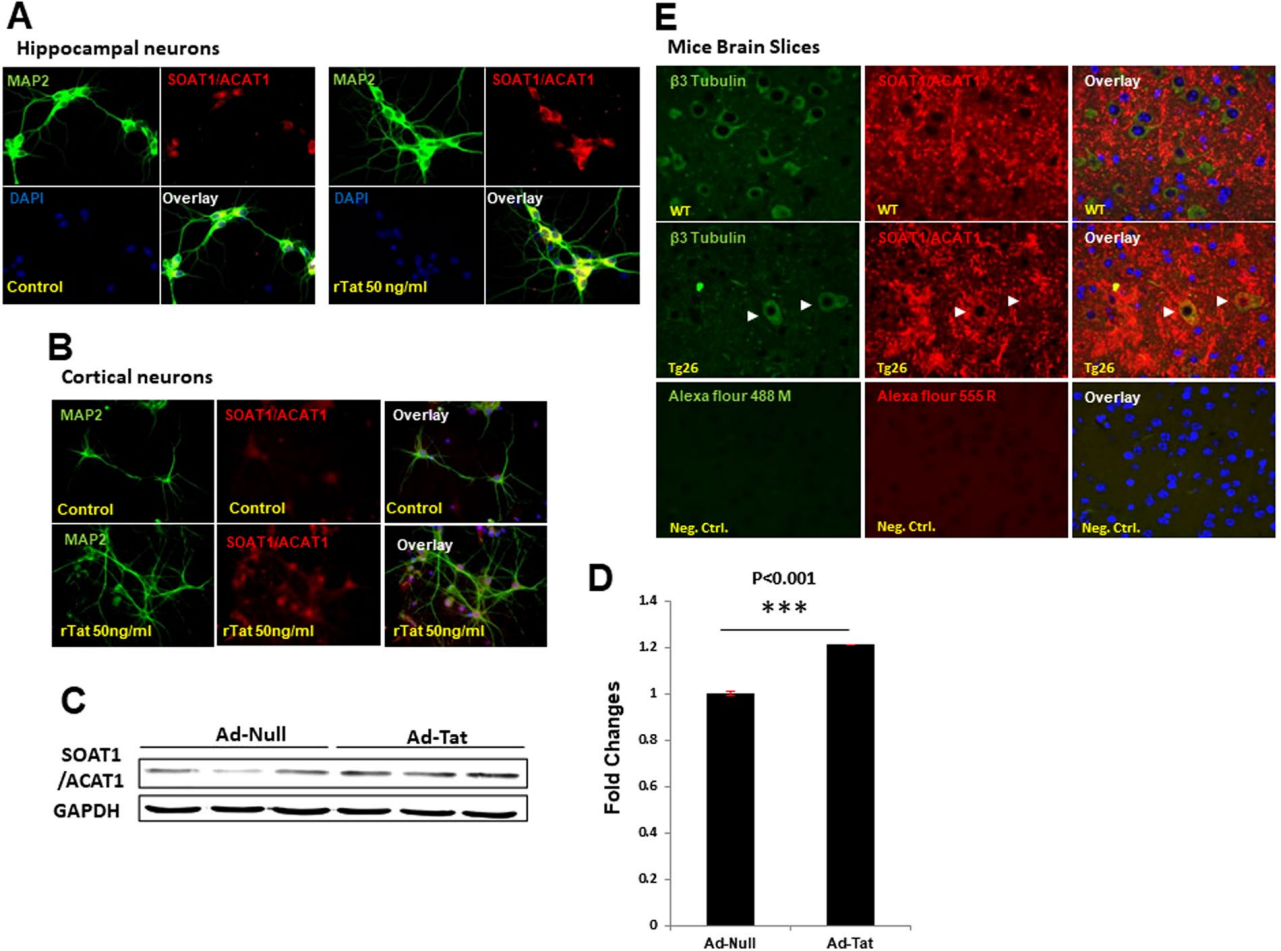
Expression of SOAT1/ACAT1 protein in the presence of HIV-1 Tat. (**A**) Immunocytochemical images of SOAT1/ACAT1 upregulation in rat hippocampal neurons exposed to 50 ng/ml rTat labeled with MAP2 (green) and SOAT1/ACAT1 (red) antibodies. (**B**) Immunocytochemistry images of SOAT1/ACAT1 upregulation in rat cortical neurons exposed to 50 ng/ml rTat labeled with MAP2 (green) and SOAT1/ACAT1 (red) antibodies. (**C**) Representative western blot and quantification of rat cortical neurons immunoblotted with anti-SOAT1/ACAT1 antibodies shows upregulation in response to Tat expression. GAPDH was used as a loading control. (**D**) Quantification of Western blot, n = 3, ^*^p < 0.0001 (via t-test) (**E**) Immunohistochemistry on formalin fixed paraffin embedded slides for Tat-transgenic mouse (TG26) and wild type mice brain tissue confirmed *in vivo* upregulation of SOAT1/ACAT1 in TG26 (negative control for each panel have been shown in this figure).

To further verify altered gene expression patterns in neurons exposed to Tat, cocaine, or Tat/cocaine, a lipoprotein signaling and cholesterol metabolism qRT-PCR array consisting of genes involved in lipid metabolism was utilized. The qRT-PCR array assessed genes within five key pathways, including low density lipoprotein (LDL) and LDL receptors, high density lipoprotein (HDL), cholesterol transport, and cholesterol metabolism pathways. Results indicated the upregulation of 13 of the 84 genes measured (Table S2). Interestingly, the upregulated genes were primarily related to the cholesterol metabolism pathway (Fig. 2A). To further validate results from the qRT-PCR array, 3 of the 13 upregulated genes that showed more than 4-fold changes in expression, specifically LRP12 (an LDL receptor gene), SORL1 (important for amyloid precursor protein trafficking) and SOAT1/ACAT1 (important for synthesis of esterified cholesterol) and were assessed by qRT-PCR (Fig. 2C). qRT-PCR results confirmed the upregulation of these genes in primary rat cultures transfected with adenovirus mediated Tat (Ad-Tat) expression or exposed to recombinant Tat (rTat) (Fig. 2C,E). Given the pivotal role of the SOAT1/ACAT1 gene in cholesterol esterification as well as its important role in the progression of neurodegenerative diseases, such as AD and Parkinson’s disease, RNA and protein measurement validation experiments were focused on SOAT1/ACAT1. Our qRT-PCR analysis revealed a significant increase (p < 0.001) in SOAT1/ACAT1 in neurons exposed to Ad-Tat or rTat versus untreated control (Fig. 2D,E). Interestingly, in rat primary neurons, we did not observe any changes in SOAT1/ACAT1 expression levels when Ad-Tat/cocaine were applied together (Fig. 2D). To confirm that these changes occurred at protein levels as well, immunocytochemical analyses of rat primary hippocampal and cortical neurons showed Tat-mediated SOAT1/ACAT1 increase respectively (Fig. 3A,B). Increased protein levels of SOAT1/ACAT1 in cortical neurons was also confirmed via western blotting (Fig. 3C). An approximate increase of 25% in SOAT1/ACAT1 protein levels were observed after Tat exposure (p < 0.001) (Fig. 3D). Similarly, cortical tissue from Tg26 transgenic mice (Fig. 3E), as well as frontal cortex tissues from the SIV (Simian immunodeficiency virus) infected macaque revealed higher expression levels of SOAT1/ACAT1 (Fig. S5).

To confirm that higher expression of SOAT1/ACAT1 was observed in the brains of HIV patients, we inquired if changes in mRNA in brain tissues from age-matched HIV- controls, HIV + ART naïve and HIV + ART-treated brains were reported using the raw microarray data publicly available on the NCBI GEO (Fig. S6A). These results indicated that significantly (p = 0.0003) higher mRNA levels of SOAT1/ACAT1 were present in HIV + ART naïve brains compared to age-matched controls or HIV + ART + samples. However, cocaine did not directly alter the SOAT1/ACAT1 mRNA expression levels in the rat neurons (Figs 2D and S6D).

### Effect of Tat and cocaine treatment on the neuronal cholesteryl esters and free cholesterol

**Figure 4:**
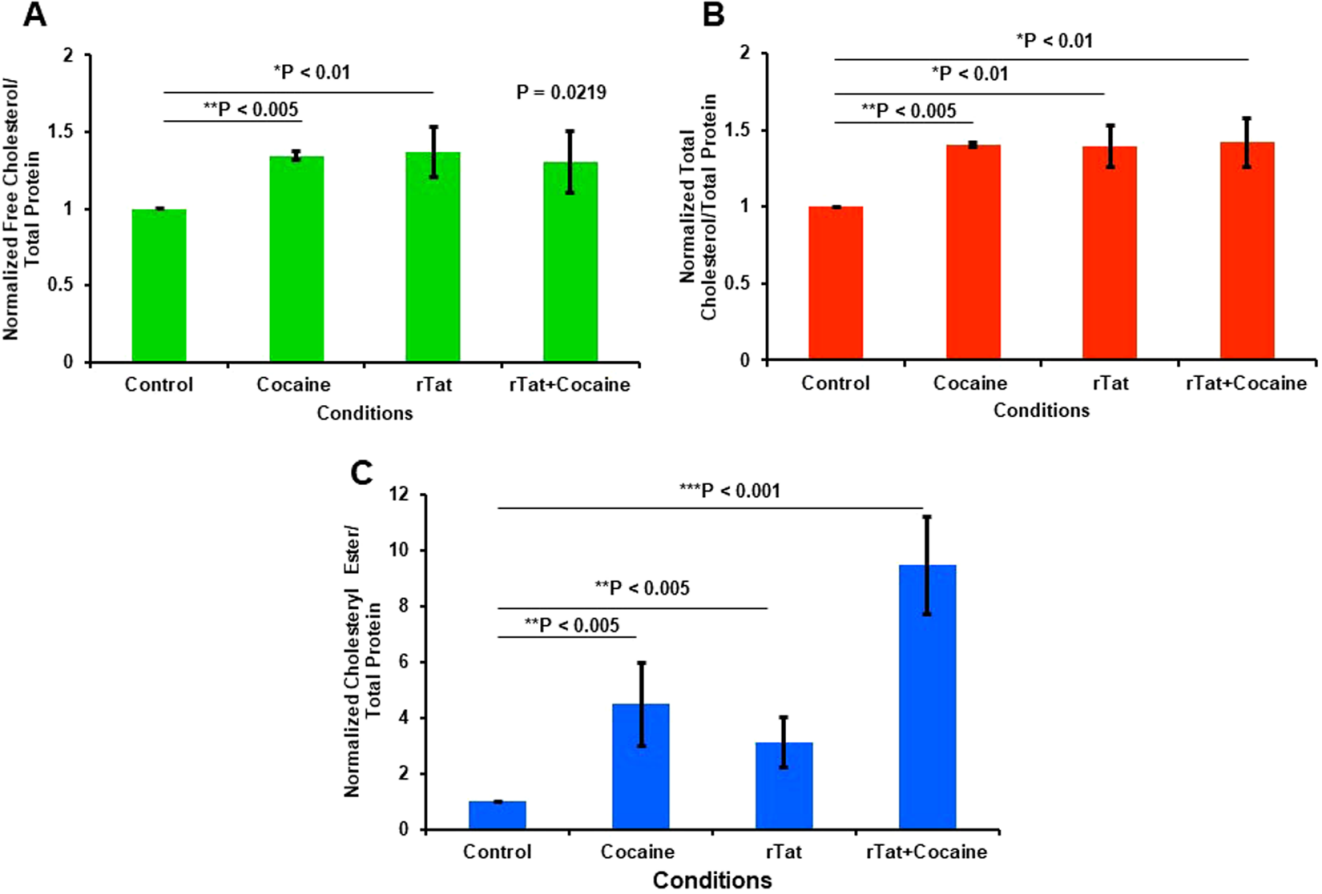
Neuronal cholesterol synthesis is induced by cocaine, Tat and Tat/cocaine. (**A**) Normalized free cholesterol levels following cocaine, Tat and Tat/cocaine treatments exhibit an average increase greater than 50% in rat neurons. (**B**) Normalized total cholesterol levels as increased by Tat and cocaine. The total cholesterol includes free cholesterol and cholesteryl esters. (**C**) Normalized cholesteryl esters in neurons exposed to cocaine, Tat and Tat/cocaine indicate an additive effect of Tat plus cocaine on the cholesteryl ester amount. The statistical analysis was conducted via t-test, n = 4.

Intercellular cholesterol levels were measured in rat primary neurons exposed to recombinant Tat, cocaine and Tat/cocaine. Cocaine, Tat and Tat/cocaine increased levels of free cholesterol, total cholesterol and cholesteryl esters (CE), respectively (Fig. 4A–C). The levels of increase in free cholesterol and total cholesterol are less than 50%; however, in cocaine, Tat and Tat/cocaine treated neurons, CE were increased 4, 3- and 9-fold, respectively (Fig. 4C). These results correlate with the activating effect of Tat and cocaine on the gene expression of cholesterol biosynthesis pathways (Fig. 7S).

## Discussion

We investigated HIV-1 Tat-, cocaine- and Tat/cocaine-mediated changes in the neuronal transcriptome through applying RNA next generation sequencing. Our findings revealed significant changes in multiple neuronal pathways important for neuronal survival and proper functioning. The results showed dysregulation in the lipid pathway in neurons, which overlaps with functional signaling changes reported in AD^45^. Previous studies have investigated changes in the global transcriptome of HIV-1 infected T-cells, B-cells, macrophages and the whole brain. In these studies, functional annotation of DEGs revealed HIV-1 adversely affects the TCA cycle, TGF-β signaling, reductive carboxylate cycle, cytokine-cytokine receptor interaction, cell cycle, DNA repair, apoptosis, immune response pathways and JAK-STAT signaling in PBMCs, CD4+ T cells and monocytes^22,47^. RNA-sequencing of HIV-1 infected monocytes and gene ontology analyses uncovered some of the HIV-1 infection specific effects on gene expression and chromatin remodeling^23^. Others have shown changes in apoptosis, cell cycle, lipid metabolism, proteasome function, protein trafficking, transcriptional regulation pathways, toll-like receptor signaling, cytokine–cytokine receptor interactions and cell cycle regulation^48,49^. Moreover, HIV-1 infection altered distinct signature pathways in the brains of HIV-transgenic rats including B-cell translocation gene (BTG) family-mediated cell cycle regulation and toll-like receptor signaling in the prefrontal cortex, and JAK/STAT signaling and eukaryotic initiation factor 2 signaling in the hippocampus and striatum, respectively^29^.

SOAT1/ACAT1 is an enzyme localized in the endoplasmic reticulum that synthesizes CE from free cholesterol and fatty acids and has an essential role in the aggregation of β-amyloid in the CNS. SOAT1/ACAT1 overexpression results in the production of excessive amounts of hydrophobic cholesteryl esters, which in turn, may lead to β-Amyloid (Aβ) deposition from the cleaved amyloid precursor protein (APP)^50^. In an animal study, SOAT1/ACAT1 KO mice on a high fat diet (HFD) showed improved cognitive functioning, higher insulin tolerance, less pro-inflammatory cytokines (including TNF-α and IL-6) and attenuated overexpression and phosphorylation of GFAP caused by HFD compared to wild type^51^. It has also been shown that SOAT1/ACAT1 KO or inhibition increases phagocytic uptake of Aβ-42 by microglia and enhances lysosomal autophagy in an mTOR-independent manner^52,53^. In mouse hippocampal neurons, SOAT1/ACAT1 ablation resulted in increased 24(S)-hydroxycholesterol synthesis rate and consequently rapid declines of hAPP and HMGR proteins^54^, suggesting the regulatory effect of SOAT1/ACAT1 protein on the cholesterol biosynthesis upstream proteins.

Cholesterol turn-over is tightly regulated in neurons where the majority of cholesterol exists as free cholesterol with CE constituting about 1% of the total cholesterol under normal conditions. Our RNA-seq data suggest the activation of mevalonate pathway under the Tat treatment. This activation is followed by upregulation of DHCR7 (which is essential to convert dehydrocholesterol to cholesterol) and upregulated cholesterol biosynthesis. This increase in the cholesterol level is reflected in our total cholesterol assay in the neurons undergoing Tat and Tat/cocaine treatment by about 40%. Changes in neuronal cholesterol levels also indicated that both free cholesterol and CE contribute to the elevation of total cholesterol. Tat-mediated cholesterol upregulation may indicate neuronal attempts to repair the cellular membrane or synaptic loss caused by Tat uptake^55^. Our RNA-Seq and qRT-PCR array also suggest Tat-induced upregulation of sterol regulatory element‐binding proteins (SREBPs) mRNAs required for regulation of the lipid and cholesterol biosynthesis genes (data not shown) as well as Lipoprotein receptor-related protein (Lrp1) gene. Lrps are of key importance in the transport of lipids and signaling. Also Lrp1 has been recognized as the main gateway of HIV-1 Tat endocytosis in neurons^56^. On the other hand, this upregulation may reflect the Tat-specific function to recruit the cholesterol metabolism pathway and provide the cholesterol required for capsid formation as well as virus replication, even though HIV does not infect neurons. Viral replication-mediated formation of CEs is implicated by some studies. For example, *in vitro* infection of hepatitis C virus has been linked to the upregulation of ACAT1 and ACAT2 genes and elevated CE synthesis, hypothesized to be a main component in lipoviral particles^57^. Our findings correlate with previous studies showing the accumulation of cholesterol in monocytes infected with HIV^58^ and HIV-induced foam cell formation through upregulation of TNF-α^59^. TNF-α upregulation is known to positively regulator SOAT1/ACAT1 expression^60^. Our observations demonstrate the upregulation of SOAT1/ACAT1 at the mRNA level in Tat-treated rat neurons, Tg26 mice and SIV-infected macaque brains. Our analysis of the microarray data published on the NCBI Geo platform^61^ confirms the SOAT1/ACAT1 mRNA overexpression of both ART-naïve and ART-treated HIV^+^ patients. Furthermore, our immunoassays show upregulation of SOAT1/ACAT1 protein in Tg26 transgenic mice and SIV macaques. Moreover, SOAT1/ACAT1 upregulation corresponds to the CE increase in neuronal cultures exposed to cocaine (>4 fold), Tat protein (>3 fold) and Tat/cocaine (>9 fold). Multiple reports have linked the Aβ plaque formation and deposition with elevation in CE^54,62^. Our observations also suggest Tat-mediated accumulation of Aβ in neurons (Fig. S6B,C). Our qRT-PCR data showing the upregulation of SORL1 (a member of LDLR family) and LRP12 may reflect an adaptive neuronal response to the accumulation of Aβ induced by increased CE formation. Therefore, Tat-mediated upregulation of cholesterol biosynthesis in neurons and subsequent induction of CE production may support a novel mechanism describing the amyloid fibril deposition repeatedly reported in HIV infection^63^.

**Figure 5:**
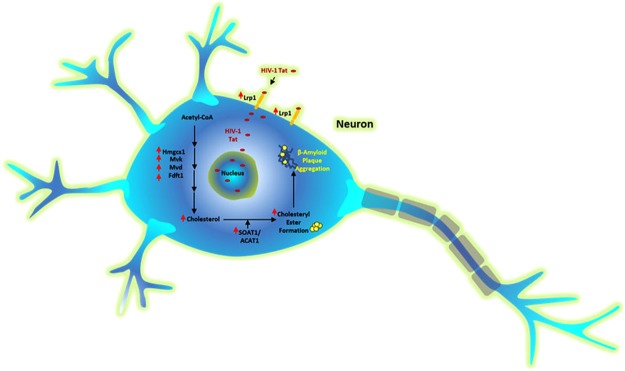
Schematic showing the upregulation of cholesterol biosynthesis genes as a result of HIV-1 Tat according to RNA-Seq data in neurons. Tat activates mevalonic acid and cholesterol esterification genes including SOAT1/ACAT1 leading to the accumulation of cholesteryl ester in neurons. Other gene symbols are as following: Hmgcs: hydroxymethylglutaryl-CoA synthase; Mvk: mevalonate kinase; Mvd: mevalonate diphosphate decarboxylase; Fdt1: farnesyl-diphosphate farnesyltransferase 1; Lrp1: Low density lipoprotein receptor-related protein 1.

Dysregulation of lipid metabolic pathway by HIV-1, as it relates to sphingolipid and ceramide, has been extensively investigated and the outcome suggested that overproduction of ceramide could eventually lead to neuronal cell injury and death^41,64–66^. Interestingly, the role of sphingolipid metabolism in the neuropathogenesis of AD via ApoE4 was also demonstrated, suggesting the importance of lipid metabolism in neurodegenerative diseases ranging from HIV-1 dementia to AD^67,68^. Here, we identified another pathway that provides a new insight to HIV-1 neuropathogenesis and age-related changes observed in HIV-associated neurocognitive disorders (HAND). Figure 5 provides a schematic of the model proposed by this research. HIV-1 Tat and cocaine promote the upregulation of cholesterol biosynthesis genes, subsequently leading to the elevated amount of free cholesterol. Further upregulation of the SOAT1/ACAT1 gene and protein results in the formation of excess CEs and amyloid-plaques which eventually contribute to the Tat/cocaine-mediated neuronal injury.

## Materials and Methods

### Ethics Statement

Guidelines regarding the care and use of laboratory animals were applied during all stages of the experimental procedures. The Temple University Institutional Animal Care and Use Committee approved all experimental protocols. In one series of experiments we used HIV-1 transgenic mice, Tg26, as described previously^69,70^.

### HIV transgenic mice - Tg26

The HIV Tg26 transgenic is a well-described mouse model which is hemizygous, encoding the entire pNL4–3 HIV-1 genome with deletion of a segment of gag/pol genes and expresses viral proteins such as Tat and Nef ^71^. The Tg26 mice pathology is similar to HIV-associated symptoms^72^.

### Macaque model

Indian rhesus macaques (Macaca mulatta) were inoculated intravenously with SIVmac251 viral swarm (5 ng p27; Tulane National Primate Research Center’s [(TNPRC; Covington, LA) Viral Core] and subsequently CD8-depleted through administration of 10 mg/kg of anti-CD8 antibody subcutaneously at 6 days post-infection (dpi) and 5 mg/kg of antibody intravenously at 8 and 12 dpi (Nonhuman Primate Reagent Resource)^59^. The SIV+ animals were sacrificed according to humane endpoints consistent with the recommendations of the American Veterinary Medical Association (AVMA) Guidelines for the Euthanasia of Animals. The development of simian AIDS was determined post-mortem by the presence of Pneumocystis carinii-associated interstitial pneumonia, Mycobacterium avium-associated granulomatous enteritis, hepatitis, lymphadenitis, and/or adenovirus infection of surface enterocytes in both small and large intestines. For ART treatments, animals were not only SIV-infected and CD8 depleted, but also received a triple ART regimen of Raltegravir (22 mg/kg oral twice daily, Merck), Tenofovir (30 mg/kg subcutaneous once daily, Gilead), and Emtricitabine (10 mg/kg subcutaneous once daily, Gilead) at 21 dpi until the timed sacrificed at 118–120 dpi. Animals were anesthetized with ketamine–HCL and euthanized by intravenous pentobarbital overdose. Animals used in the study were housed at the TNPRC. All animals used in this study were handled in strict accordance with American Association for Accreditation of Laboratory Animal Care with the approval of the Institutional Animal Care and Use Committee of Tulane University.

### Rat embryonic cell culture and treatments

Embryonic day 18 (E18) rat hippocampal neurons were obtained from the Comprehensive NeuroAIDS Center (CNAC) and prepared and maintained as described previously^20^. After 10 days in culture, neurons were treated with 50 ng/ml recombinant HIV-1 Tat protein or adenovirus (MOI: 1) expressing Tat as well as 2 µM cocaine in sodium citrate (pH 5.0), which was repeated every 24 hours for a total of 72 hours^20^. Neurons were exposed to rTat (101, Immunodiagnostics Inc, MA, 50 ng/ml), Cocaine hydrochloride (2 μM, Sigma-Aldrich, St. Louis, MO) and rTat/cocaine for 48 hours. Negative controls received no treatments or were transduced with adeno-null (Ad-null). Following the treatments, total RNA was prepared using Trizol (ThermoFisher, Carlsbad, CA) according to the manufacturer’s protocol. RNA quality was determined on an agarose gel. Neurons were harvested after 72 hours for RNA isolation and qRT-PCR validations. RNA-Seq analysis was performed at Applied Biological Materials Inc. (British Colombia, Canada) and with 80 million 2 × 75 bp paired-end reads.

### RNA isolation and cDNA preparation

Total RNA was isolated using the Direct-zol RNA prep mini kit (Zymo Research, CA, USA) following the manufacturer’s protocol. cDNA synthesis on total RNA samples was performed with High Capacity cDNA Reverse Transcription Kit (Thermo Fisher Scientific) according to manufacturer’s protocol.

### PCR Arrays

The amplified cDNA was added to the RT2 qRT-PCR SYBR green Master Mix (QIAGEN) following the array protocol. Real-Time PCR was performed on the LightCycler® 96 (Roche) and used SYBR green detection with the following thermal profile: segment 1–1 cycle: 95 °C for 10 minutes, segment 2–40 cycles: 95 °C for 15 seconds followed by 60 °C for 1 minute. All data from PCR was collected by the LightCycler® 96 Instrument Software (Version 2.0, May 2013, Software Version 1.1). To validate the result of targeted genes, primers were designed considering the exons junction positions in the genomic DNA, primer specificity and efficiencies checked by RT-PCR using Q5 High-Fidelity PCR Kit (City, State, New England Biolabs). All qRT-PCR reactions were performed with the LightCycler® 96 and Roche cyber green qRT-PCR Master Mix buffer according to manufacturer’s protocol. Relative quantification was performed via Actin primer reference gene normalization. Primer sequences are shown below (Table 2).

**Table 2:** The sequence of forward and reverse oligos designed for amplifying target genes in rat and human.

Gene	Forward primer	Reverse primer
Rat-SOAT1	CCAGTGCTCTTCGTGCTCTTC	ACGTACCGACAAGTCCAGGT
Rat-Sorl1	ACCTGATGTGTGACTGCC	CGAGTGGCCATTGTGGCAT
Rat-Lrp12	CTTCAGACTACCCCGCCAAG	AACTTTGGTGAAGCGGGACA
Human-SOAT1	GAATGGTATGCACGTCAGCACT	CAAGTGACCTAGGAATGGAGTGG
Human-Sorl1	GCCATCCTGTACACGAAGCAC	GTATGCTGATGGGTCCACTCC
Human-Lrp12	CATAGGGAGCCTCATCTGTGG	CGATACTGTGCATCTGTGTGC

### Analysis of real-time PCR array data

Each array contained six separate housekeeping genes (Actb, B2m, Hprt1, Ldha, Rplp1, RGDC) that were used for normalization of the sample data. Ct data were entered into the QIAGEN PCR array Data Analysis Web Portal to perform quantification and calculate fold changes using the ΔΔ Ct method^73^. Any Ct value >35 was considered to be negative. If the Ct value of the genomic DNA control was >30, then no genomic DNA was detectable.

### Isolation of protein and Western blot analysis

Neurons were washed with PBS and lysed in RIPA buffer (50 mM Tris–HCl, pH 7.5, 150 mM NaCl, 0.5% NP40, 1:100 protease inhibitor cocktail, Calbiochem, San Diego, CA). Cell debris and insoluble particles were removed by centrifugation and protein concentration was determined by the Bradford method (BioWorld, Columbus, OH). Equal amounts of proteins were separated on SDS-PAGE and transferred to Odyssey nitrocellulose membrane (Li-Cor, Lincoln, NE) by wet transfer (Bio-Rad, Philadelphia, PA). The following primary antibodies were used for Western blotting: anti-SOAT1/ACAT1 (1:1,000, Proteintech, Rosemont, IL), anti-GAPDH (1:2,000, Santa Cruz, sc-32233).

### Immunolabeling and microscopy

Primary neurons in two-well chamber slides were washed with PBS and fixed in 4% Paraformaldehyde for 15 min. Neurons were then washed three times with PBS and permeabilized with 0.1% triton X100 in PBS for 5 min, followed by washing three times with PBS. Neurons were blocked at room temperature for 1 hour in blocking buffer (10% FBS, 0.1% triton X100 in PBS). Neurons were subsequently incubated with primary antibodies in blocking buffer at 4 °C overnight. Detection of labeled proteins was conducted via Alexa Flour (1:1,000, ThermoFisher Scientific) in blocking buffer for 1 hour at room temperature. After mounting slides with VECTASHIELD hard set mounting medium with DAPI (Vector Laboratories, Burlingame, CA), images were captured using a Leica microscope system. Formalin fixed paraffin embedded brain tissues from mouse (30 days old) and SIV- control and SIV+ macaques sectioned at 5um following standard protocol. Briefly, after dewaxing, sections were placed in boiling water plus 1% antigen unmasking solution. Mouse sections were probed with anti-SOAT1/ACAT1 (1:200) and anti- β3-tubulin (1:500). Macaque sections were probed with anti-SOAT1/ACAT1 (1:200) and β3-tubulin (1:500). The fluorescent labeling followed the same procedure as described for immunocytochemistry.

### Free and total cholesterol and cholesteryl ester quantification

E18 rat primary neurons were cultured in 12-well plates (10^6^/well) and were maintained 12 DIV (days *in vitro*) prior to the treatments. Recombinant Tat protein and cocaine (dissolved in sodium citrate buffer, pH = 5.5) were added at final concentrations of 50 ng/ml and 4 μM, respectively. This treatment was repeated every 24 hours for a total of 72 hours (15 DIV) following which cells were harvested using chloroform/isopropanol/NP-40 buffer. After solvent removal, the extracted lipid droplets under the control, cocaine, Tat and Tat\cocaine were used to determine the free and total cholesterol and CE according to manufacturer’s instructions using the cholesterol quantification kit (Sigma-Aldrich, St. Louis, MO).

### RNA-Seq data acquisition and alignment

Short-reads were obtained through an Illumina® next generation sequencing facility (Novogene, Chula Vista, CA), with each library including 80 million single-end reads of 75 bp length. Short-reads were aligned versus the reference genome of rat (Rattus Norvegicus Rnor_6.0.85) with the corresponding annotation file in the gtf format, both obtained from NCBI. The alignment utilized Bowtie algorithm with the following parameters: maq error of 70, seed length of 28 and maximum backtracks of 800. The computational capacity was provided by a dual-Xeon CPU computer benefitting from CUDA parallel computing platform implemented using NVIDIA® Tesla M2090 and Quadro FX 580.

### Probe definition and expression profile analysis

After aligning the short-reads, the resulting BAM files were exported into the SeqMonk® environment, where the relative abundance of reads was computed for the defined probes (genes). The probes were defined according to the gtf annotation file. For the probe generation step, we considered the feature probe generator and designed the probe sets for the mRNAs. Each probe represented the gene’s exons where exact duplicates were removed. For the quantification step, the read count quantification scheme was adopted. The reads were counted on the opposite strand of the probe and the total read count were corrected per millions of reads and the reads only were counted within the probes. After counting the number of reads, this number was normalized to the probe length (in base pairs). For the visualization and analysis purposes, log_2_ of the computed normalized read counts was calculated.

### Hierarchical clustering

Hierarchical clustering was performed using the mRNA read count data. The outcome of this analysis was the presence of different clusters under control, Tat, cocaine and Tat/cocaine. The groups (clusters) were represented using a dendrogram, where the members of each cluster are of higher similarity with each other than the members of other clusters. This analysis provides information on how the expression profile of a certain gene is compared to the rest of the genes and whether there are similarly expressed genes which may lead to the conclusions of the relationships of the genes within each cluster.

### MA plot generation

To capture the expression variations in each pair, ${M}_{X,Y}={\mathrm{log}}_{2}{{\rm{RPKM}}}_{Y}-{\mathrm{log}}_{2}{{\rm{RPKM}}}_{X}$ and ${A}_{X,Y}=\frac{1}{2}({\mathrm{log}}_{2}{{\rm{RPKM}}}_{Y}+{\mathrm{log}}_{2}{{\rm{RPKM}}}_{X})$ were calculated and plotted versus each other, where X and Y denote two experimental conditions. This approach allows the application a fold change threshold, for example 2, which can identify the ratio of the genes that show 2 fold up- or down-regulation.

### Correlation network analysis

The Spearman’s rank correlation was used to derive the correlation between the expression profiles of genes of interest with respect to each other. This analysis provided a framework to identify genes showing the most similar response to the input conditions, helping to determine the groups of inter-related genes for the validation step. In this analysis, we used this measure to derive a group of genes that show the highest (absolute value of 0.995) with our target genes at the mRNA expression level.

### Functional annotation and gene ontology

To further examine the impact of Tat- and cocaine-mediated changes in the neuronal transcriptome and detect impairments in the neuronal pathways, the expression profiles of the whole transcriptome were supplied to the pathway analysis algorithms in the AltAnalyze®^74^. environment. The algorithms were supplied with multiple databases such as KEGG and WikiPathways to obtain the most updated versions of the metabolic and regulatory pathways. As an output, the software package generated a list of regulatory pathways based on the gene’s annotations which were most significantly impacted by the experimental conditions. Gene ontology (GO)-based functional annotation and pathway analysis of overrepresented genes and visualization were performed using the Cystoscope applications of Bingo and EnrichmentMap^75^. Two types of approaches were essentially employed to determine the input genes fed into the applications: i) based on the expression fold change and ii) based on being recognized within the same expression pattern clusters. This step was followed by detailed pathway analysis to investigate the GO-detected overrepresented pathways in PathVisio^76^ environment.

### Statistical analysis

The MathWorks MATLAB® was used to post-process the normalized read count data. The statistical toolbox was utilized to perform different tasks including clustering, correlation network analysis and visualization. t-test has been used in all our studies to compute p-values. Quantified data show mean ± StDev. The significance levels were considered as following: ^*^p < 0.01, ^**^p < 0.005, ^***^p < 0.001.

## Electronic supplementary material

Supplemental Figures and Tables
