# Supplementary material for: Dysregulation of Neuronal Cholesterol Homeostasis upon Exposure to HIV-1 Tat and Cocaine Revealed by RNA-Sequencing
Source: Sci Rep. 2018 Nov 2;8:16300. doi: 10.1038/s41598-018-34539-9 (PMC6215004; doi:10.1038/s41598-018-34539-9)
Supplement: Supplementary file 1 — Supplemental Figures and Tables [file 41598_2018_34539_MOESM1_ESM.pdf]

October 26, 2018

Dysregulation of Neuronal Cholesterol Homeostasis upon Exposure to HIV-1 Tat and Cocaine Revealed by RNA-Sequencing

SUPPLEMENTARY FIGURES

Taha Mohseni Ahooyi, Masoud Shekarabi, Bahareh Torkzaban, T. Dianne Langford,

Tricia H. Burdo, Jennifer Gordon, Prasun K. Datta, Shohreh Amini, Kamel Khalili*

Department of Neuroscience

Center for Neurovirology

Comprehensive NeuroAIDS Center

Lewis Katz School of Medicine at Temple University

3500 N. Broad Street

Philadelphia, PA 19140

*Corresponding author

Address: Department of Neuroscience, Katz School of Medicine at Temple University, 3500 N. Broad Street, Philadelphia, PA 19140

Phone: 215.707.4500; Fax: 215.707.4888; Email: kamel.khalili@temple.edu

Supplementary Figures and Tables

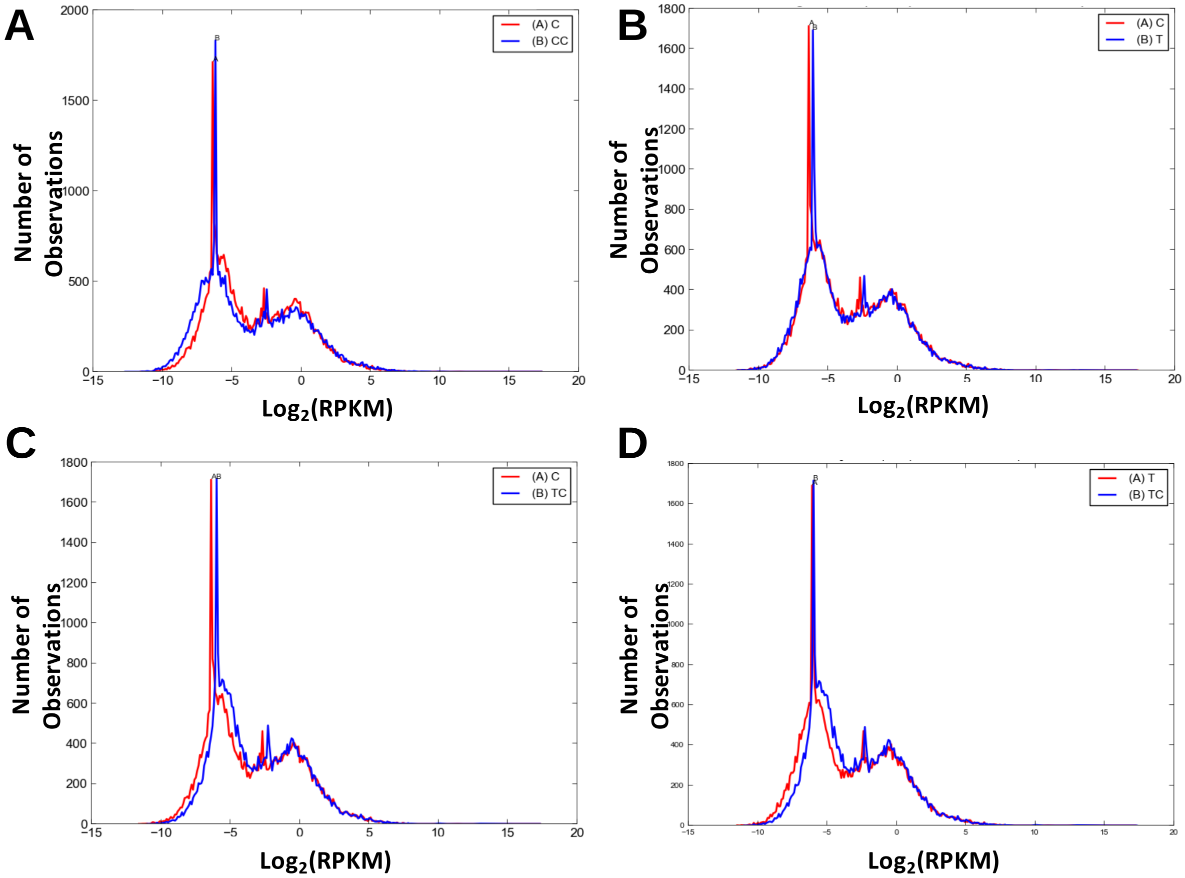

Figure S1: Pairwise comparison of genome-wide number of observations vs. log_2(Expression). **A**. control and cocaine, **B**. control and Tat, **C**. control and Tat/cocaine and **D**. Tat and Tat/cocaine. C: control, T: Tat, CC: cocaine and TC: Tat/cocaine.

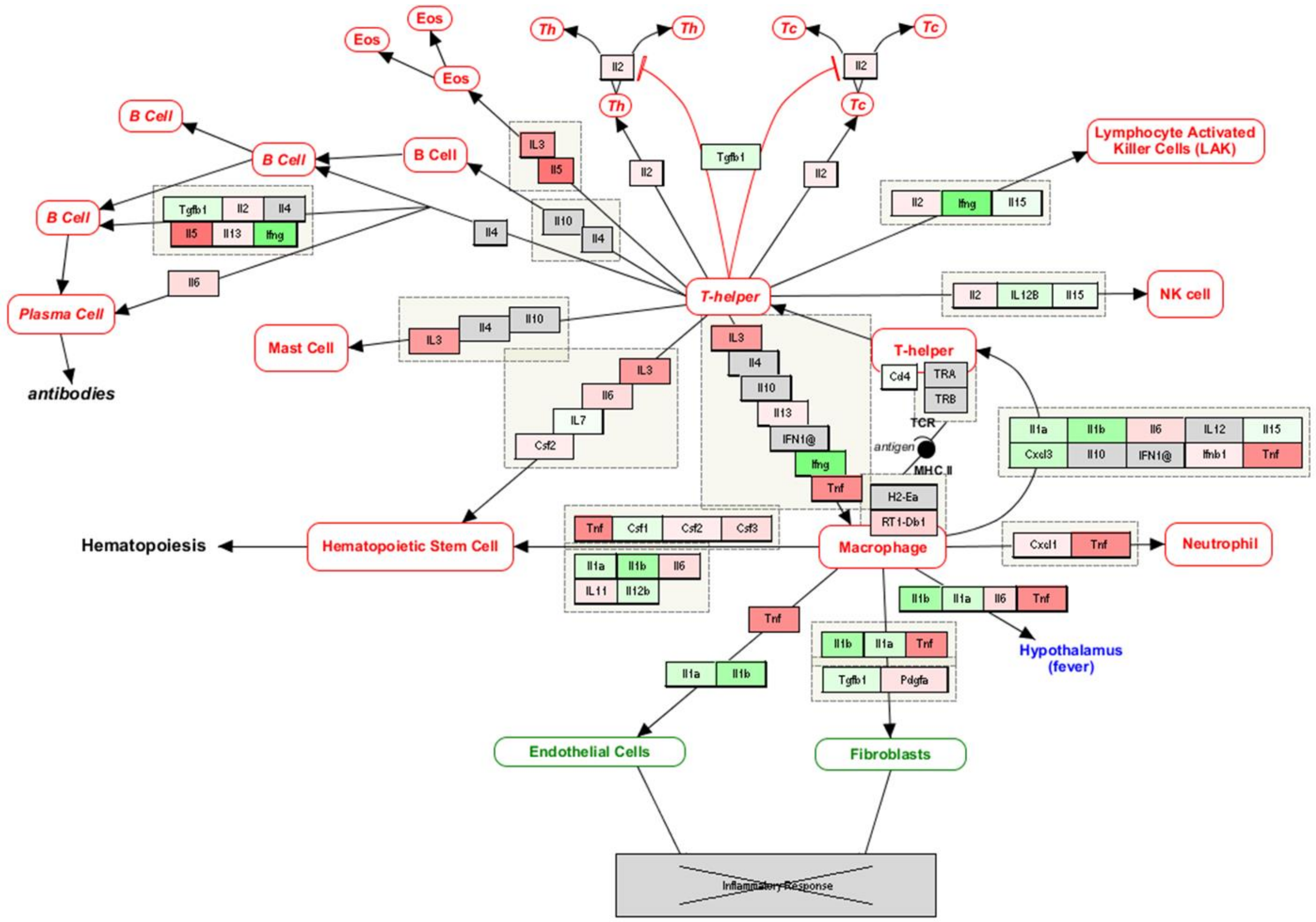

Figure S2: Tat-induced upregulation of cytokine and inflammatory response pathway. Up- and downregulated genes are indicated by red and green, respectively. The darker colors indicate higher upregulation or lower downregulation levels. Gray indicates genes with no expression data available.

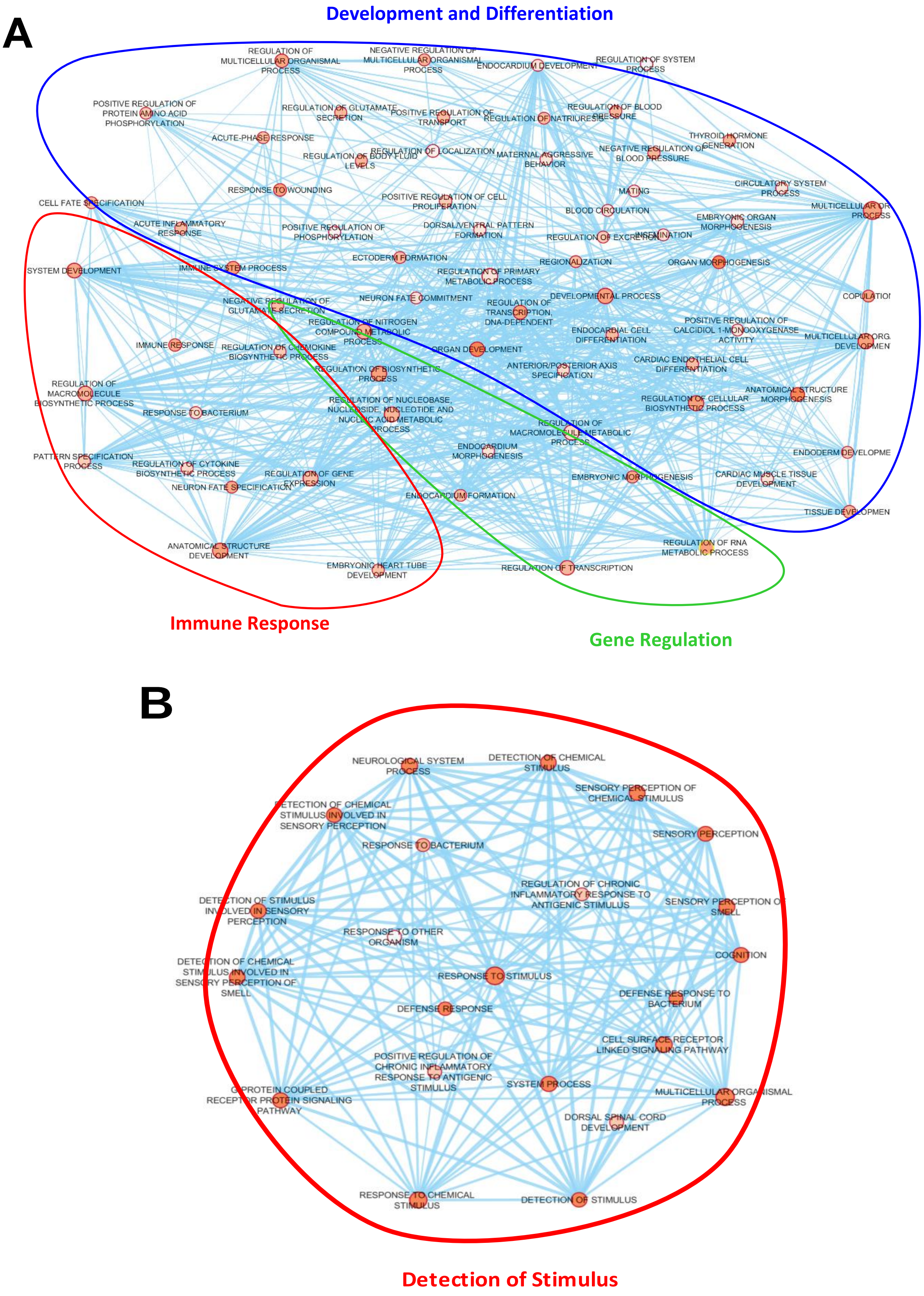
A
Development and Differentiation
Immune Response
Gene Regulation
REGULATION OF MULTICELLULAR ORGANISMAL PROCESS
NEGATIVE REGULATION OF MULTICELLULAR ORGANISMAL PROCESS
ENDOCARDIUM DEVELOPMENT
REGULATION OF SYSTEM PROCESS
POSITIVE REGULATION OF PROTEIN AMINO ACID PHOSPHORYLATION
REGULATION OF GLUTAMATE SECRETION
POSITIVE REGULATION OF TRANSPORT
REGULATION OF NATRIURESIS
REGULATION OF BLOOD PRESSURE
THYROID HORMONE GENERATION
ACUTE-PHASE RESPONSE
REGULATION OF BODY FLUID LEVELS
REGULATION OF LOCALIZATION
MATERNAL AGGRESSIVE BEHAVIOR
NEGATIVE REGULATION OF BLOOD PRESSURE
RESPONSE TO WOUNDING
MATING
CIRCULATORY SYSTEM PROCESS
CELL FATE SPECIFICATION
POSITIVE REGULATION OF CELL PROLIFERATION
BLOOD CIRCULATION
ACUTE INFLAMMATORY RESPONSE
POSITIVE REGULATION OF PHOSPHORYLATION
DORSAL/VENTRAL PATTERN FORMATION
REGULATION OF EXCRETION
INSEMINATION
EMBRYONIC ORGAN MORPHOGENESIS
SYSTEM DEVELOPMENT
IMMUNE SYSTEM PROCESS
ECTODERM FORMATION
REGULATION OF PRIMARY METABOLIC PROCESS
REGIONALIZATION
ORGAN MORPHOGENESIS
NEGATIVE REGULATION OF GLUTAMATE SECRETION
NEURON FATE COMMITMENT
DEVELOPMENTAL PROCESS
REGULATION OF TRANSCRIPTION, DNA-DEPENDENT
REGULATION OF NITROGEN COMPOUND METABOLIC PROCESS
ENDOCARDIAL CELL DIFFERENTIATION
POSITIVE REGULATION OF CALCIDIOL 1-MONOOXYGENASE ACTIVITY
IMMUNE RESPONSE
REGULATION OF CHEMOKINE BIOSYNTHETIC PROCESS
ORGAN DEVELOPMENT
REGULATION OF BIOSYNTHETIC PROCESS
ANTERIOR/POSTERIOR AXIS SPECIFICATION
CARDIAC ENDOTHELIAL CELL DIFFERENTIATION
REGULATION OF MACROMOLECULE BIOSYNTHETIC PROCESS
REGULATION OF NUCLEOBASE, NUCLEOSIDE, NUCLEOTIDE AND NUCLEIC ACID METABOLIC PROCESS
REGULATION OF CELLULAR BIOSYNTHETIC PROCESS
ANATOMICAL STRUCTURE MORPHOGENESIS
RESPONSE TO BACTERIUM
REGULATION OF MACROMOLECULE METABOLIC PROCESS
ENDOCARDIUM MORPHOGENESIS
PATTERN SPECIFICATION PROCESS
REGULATION OF CYTOKINE BIOSYNTHETIC PROCESS
REGULATION OF GENE EXPRESSION
EMBRYONIC MORPHOGENESIS
CARDIAC MUSCLE TISSUE DEVELOPMENT
NEURON FATE SPECIFICATION
ENDOCARDIUM FORMATION
ANATOMICAL STRUCTURE DEVELOPMENT
EMBRYONIC HEART TUBE DEVELOPMENT
REGULATION OF TRANSCRIPTION
REGULATION OF RNA METABOLIC PROCESS
B
Detection of Stimulus
NEUROLOGICAL SYSTEM PROCESS
DETECTION OF CHEMICAL STIMULUS
SENSORY PERCEPTION OF CHEMICAL STIMULUS
DETECTION OF CHEMICAL STIMULUS INVOLVED IN SENSORY PERCEPTION
SENSORY PERCEPTION
RESPONSE TO BACTERIUM
REGULATION OF CHRONIC INFLAMMATORY RESPONSE TO ANTIGENIC STIMULUS
DETECTION OF STIMULUS INVOLVED IN SENSORY PERCEPTION
RESPONSE TO OTHER ORGANISM
COGNITION
DETECTION OF CHEMICAL STIMULUS INVOLVED IN SENSORY PERCEPTION OF SMELL
RESPONSE TO STIMULUS
DEFENSE RESPONSE TO BACTERIUM
DEFENSE RESPONSE
CELL SURFACE RECEPTOR LINKED SIGNALING PATHWAY
POSITIVE REGULATION OF CHRONIC INFLAMMATORY RESPONSE TO ANTIGENIC STIMULUS
G-PROTEIN COUPLED RECEPTOR PROTEIN SIGNALING PATHWAY
SYSTEM PROCESS
MULTICELLULAR ORGANISMAL PROCESS
DORSAL SPINAL CORD DEVELOPMENT
RESPONSE TO CHEMICAL STIMULUS
DETECTION OF STIMULUS

C

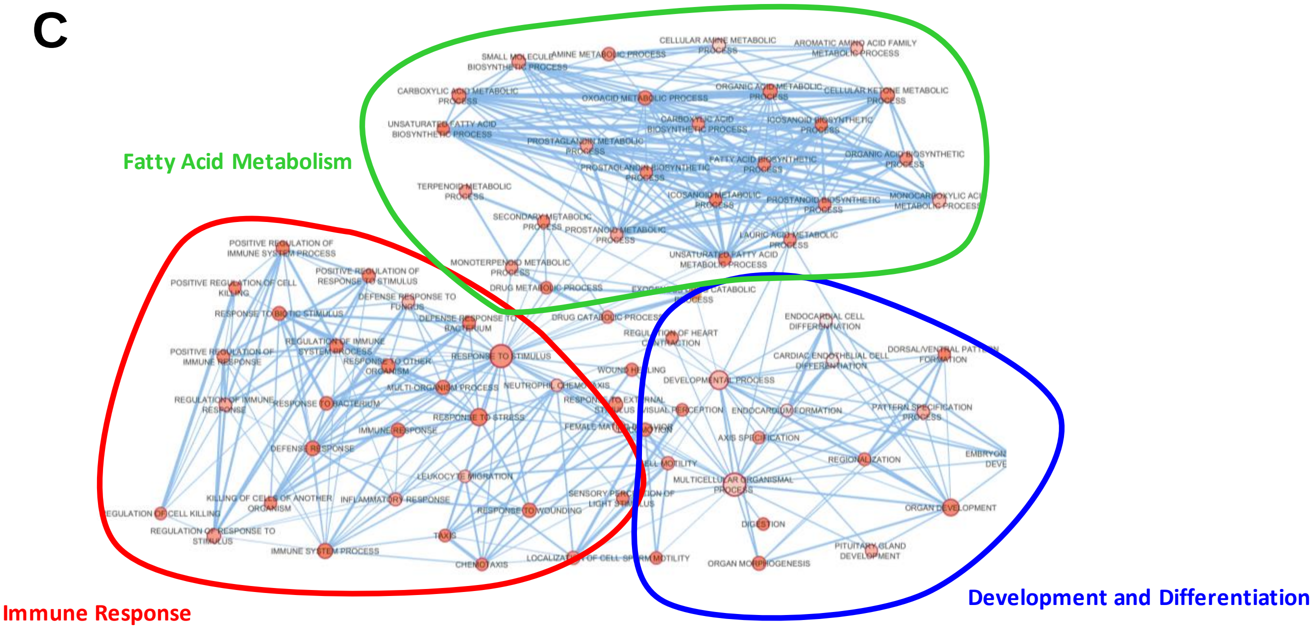

D

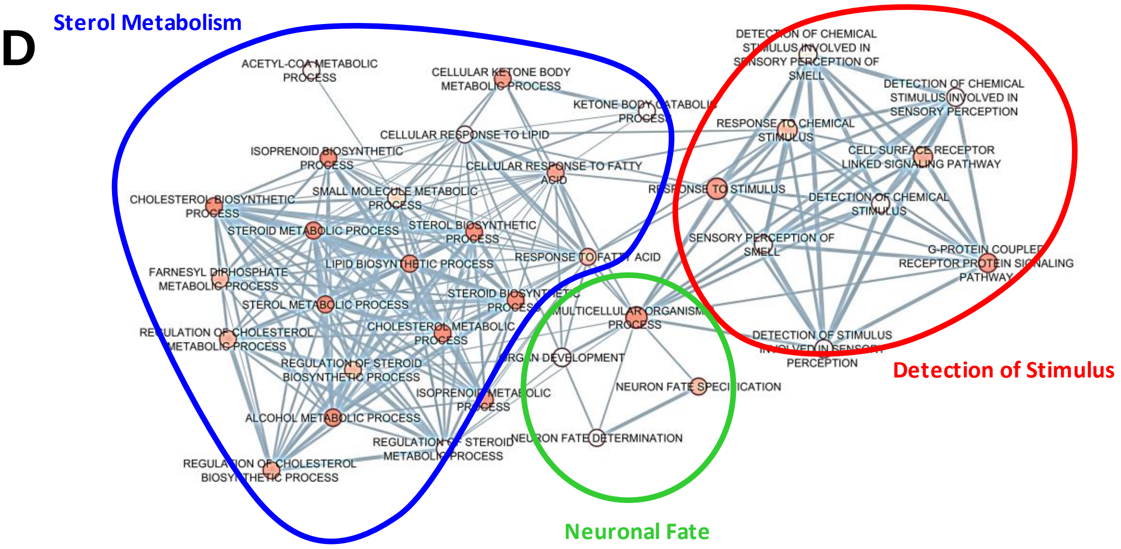

Figure S3. Detection of overrepresented pathways with at least two-fold up or down regulation. **A.** Tat downregulated pathways include differentiation and developmental pathways, gene regulatory and immune response, **B.** Tat upregulated pathways are almost entirely associated with the detection of stimulus including olfactory genes, **C.** similar to Tat, cocaine downregulated pathways include both immune response and developmental processes. In addition, cocaine shows down regulatory effects on fatty acid metabolism. **D.**

Cocaine upregulated pathways include detection of stimulus (similar to Tat), neuronal fate and sterols (including cholesterol) metabolism.

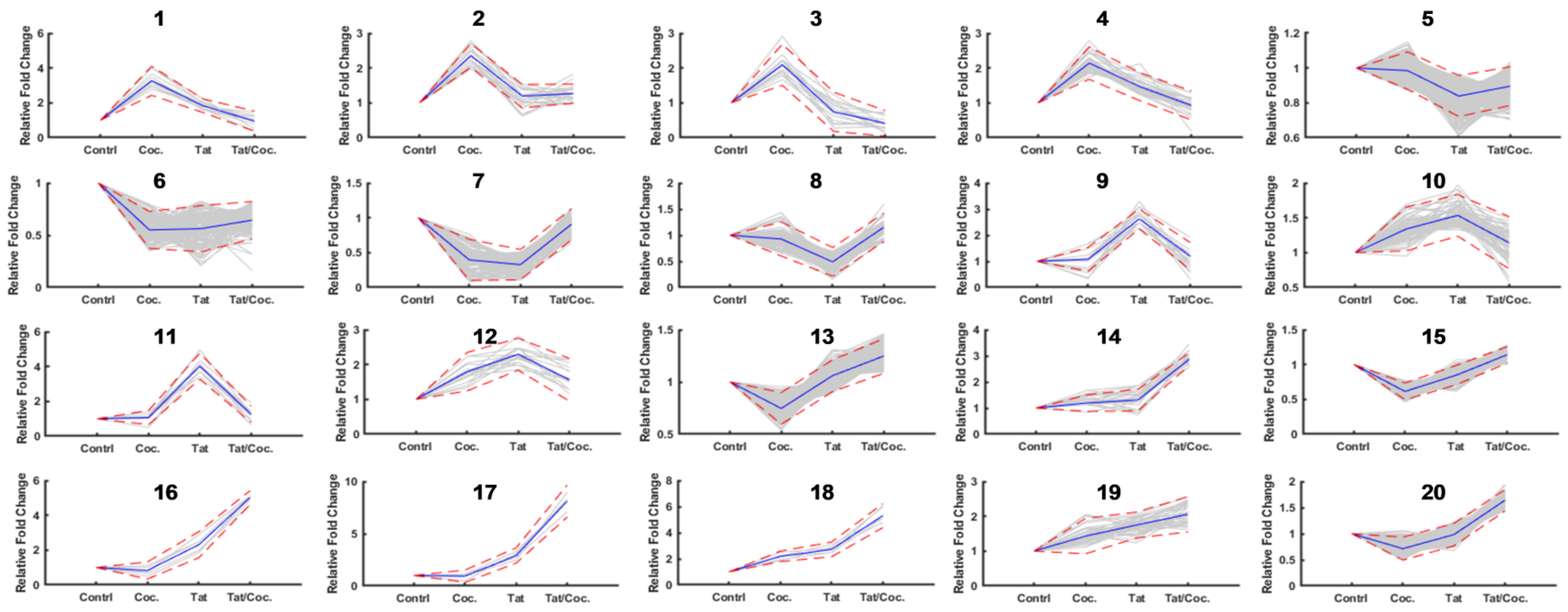

Figure S4. k-means clustering of all cellular genes to group similar differential expression patterns upon Tat, cocaine and Tat/cocaine treatments. After normalization with respect to the basal (control) gene expression levels, the quartet of (Expr(contrl), Expr(cocaine, Expr(Tat), Expr(Tat/cocaine)) of all genes were introduced the k-means clustering algorithm with k = 20. Based on the fold changes and expression patterns, the algorithm clustered the genes into 20 clusters (the average pattern shown by solid blue line and 0.05 confidence intervals shown by red dashed lines). Of our particular interest were highly upregulated and populated clusters (to minimize the functional annotation p-value) with the highest effect under Tat and Tat/cocaine (clusters 13-20). Panel #20 is detailed in Figure 2A.

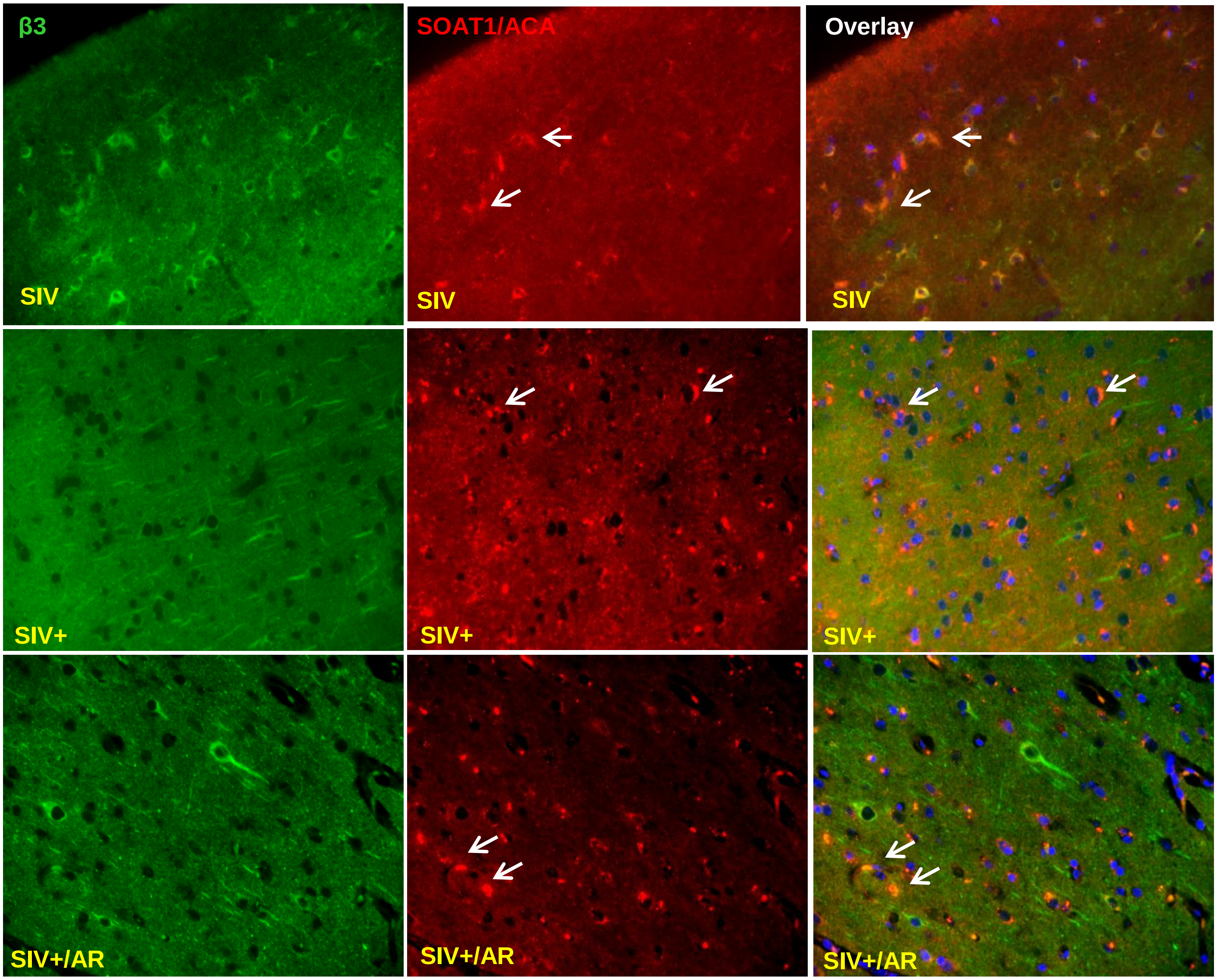

Figure S5: Expression patterns of SOAT1/ACAT1 in control (SIV-), SIV+ and SIV+/ART macaques. β3-Tubulin labels neurons (green) and ACAT1/SOAT1 is labeled in red (arrows). SIV+/ART indicate infected animals on anti-retroviral therapy.

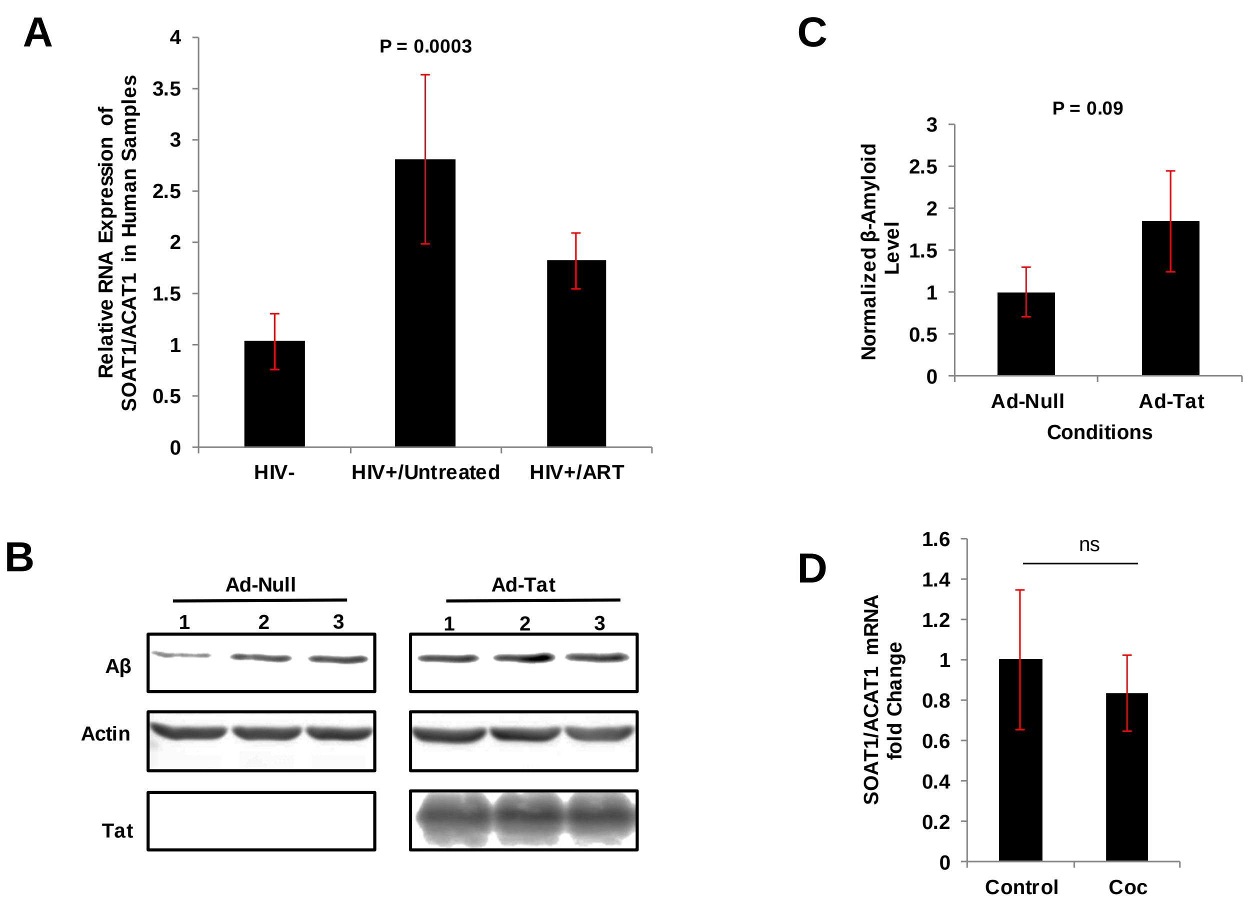

Figure S6: HIV and Tat and cocaine induced SOAT1/ACAT1 in human brain and primary neurons. A. SOAT1/ACAT1 mRNA levels in the brain tissue sections derived from autopsy of uninfected, untreated HIV-1+ and ART-treated HIV-1+ patients were analyzed from the raw microarray data publicly available on the NCBI GEO, $n \geq 6$ (Borjabad et al. 2011, GDS4231). These data show a significant increase ($P = 0.0003$, using t-test) in the SAOT1/ACAT1 level in the HIV-1 group. **B.** AdTat-induced β-Amyloid (Aβ) aggregation *in vitro* in rat primary neurons (N=3). Actin was used as a loading control. **C.** Quantification of changes in Aβ protein levels normalized to actin ($P = 0.09$) **D.** qRT-PCR data of cocaine's (coc) effect on the SOAT1/ACAT1 mRNA levels in rat primary neurons. (ns: not significant). Ad-Null, Ad-Tat were applied with MOI = 1.

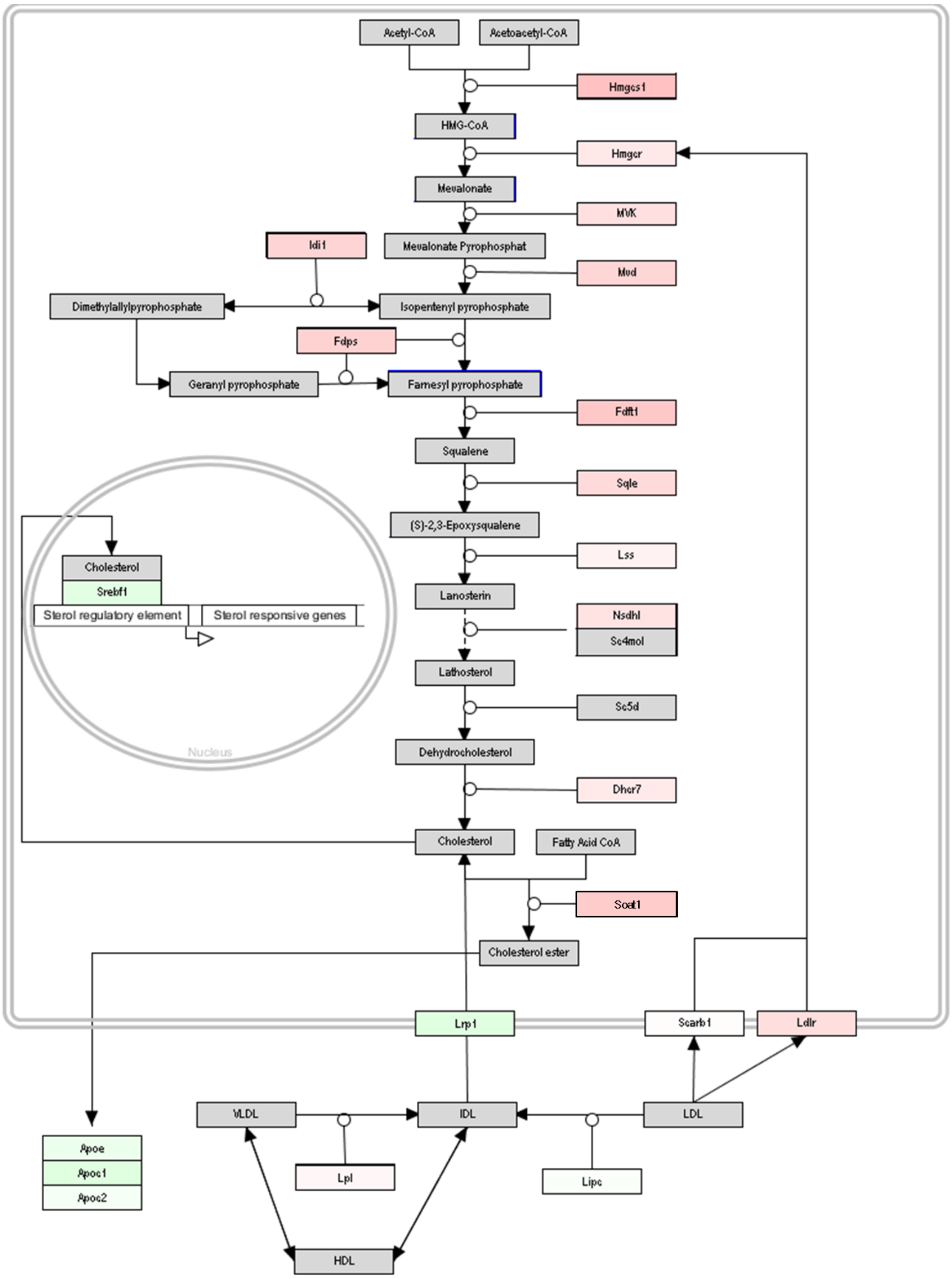

Figure S7: Tat-induced upregulation of cholesterol metabolism pathway. Genes overrepresented by the functional annotation schemes; up- and downregulated genes are indicated by red and green colors, respectively. Gray indicates genes for which no data readings were obtained.

Cocaine	Sig.	Tat	Sig.	Tat/Cocaine	Sig.
Organellar large ribosomal subunit	*	Transcription, DNA-dependent	**	Flavonol 3-sulfatransferase activity	**
Nerve growth factor receptor signaling pathway	*	DNA binding	**	Positive regulation of type IV hypersensitivity	***
Negative regulation of 1-kappaB kinase/NF-kappaB cascade	*	Pre-mRNA 3'-splice site binding	**	Positive regulation of phospholipid biosynthetic process	***
Activation of NF-kappaB-inducing kinase activity	*	Determination of left/right asymmetry in nervous system	***	Estrone sulfotransferase activity	***
Spliceosome assembly	*	Regulation of transcription, DNA-dependent	***	Zinc ion binding	***
Protein localization in plasma membrane	*	Intracellular	***	*Central nervous system development*	**
Intracellular membrane-bounded organelle	**	Zinc ion binding	***	*Neural tube closure*	**
Growth cone membrane	**	*Positive regulation of type IV hypersensitivity*	**	*Protein kinase A signaling cascade*	**
Nucleolus	**	*Positive regulation of phospholipid biosynthetic process*	**	*Vitellogenesis*	**
Pre-mRNA 3'-splice site binding	**	*Intracellular*	***	*Transcription, DNA-dependent*	***
Intracellular	***	*Positive regulation of humoral immune response*	***	*Nucleus*	***
Zinc ion binding	***	*Protein kinase C signaling cascade*	***	*Intracellular*	***
Regulation of transcription, DNA-dependent	***			*Regulation of transcription, DNA-dependent*	***
Positive regulation of humoral immune response	***			*Sequence-specific DNA binding transcription factor activity*	***
Protein kinase C signaling cascade	***				
Sequence-specific DNA binding transcription factor activity	***				
Viral reproduction	***				

Table S1: Functional annotations of the regulatory pathways most significantly affected by cocaine, Tat and Tat/cocaine as compared with control. Pathways in plain bold and bold italics typeface are down- and upregulated, respectively.

LDL Receptors & Associated Proteins	LDL Receptors	Cxcl16, Ldlr, Lrp10, Lrp12, Lrp1b, Lrp6, Olr1, Stab2, Vldlr
	LDL Receptor Associated Proteins	Lrpap1, Pcsk9, Snx17
LDL Associated Proteins		Ankra2, Apoa4, Cdh13, Colec12, Scarf1, Sorl1
HDL Associated Proteins		Apoa1, Apod, Apof, Apol2
Cholesterol Transport	Cholesterol Transporters	Abca1, Abcg1, Apoa1, Apoe, Stard3
	Cholesterol Efflux	Abca1, Abcg1, Apoa1, Apoa4, Apoe
	Reverse Cholesterol Transport	Abca1, Apoa1, Apoa2, Apoa4, Apoe, Lcat
	Other Cholesterol Transport Genes	Apob, Ldlr, Osbpl5
Cholesterol Metabolism	Cholesterol Absorption	Cel, Ldlr
	Cholesterol Catabolism	Akr1d1, Apoe, Cel, Cyp39a1, Cyp46a1, Cyp7a1, Scarf1, Snx17, Trerf1
	Cholesterol Homeostasis	Abca1, Abcg1, Angptl3, Apoa1, Apoa2, Apoa4, Apoe, Lcat, Ldlr, Ldlrap1, Pcsk9
	Cholesterol Biosynthesis	Acaa2, Cnbp, Cyb5r3 (Dia1), Cyp51, Dhcr24, Dhcr7, Ebp, Fdft1, Fdps, Hmgcr, Hmgcs1, Hmgcs2, Idi1, RGD1564999, Mvd, Mvk, Nsdhl, Pmvk, Prkaa1 (Ampk), Prkaa2, Prkag2, Tm7sf2
	Other Cholesterol Metabolism Genes	Abca2, Apob, Apoc1, Apoc3, Apof, Apol2, Cyp11a1, Cyp7b1, Cela3b, Hdlbp, Il4, Insig1, Insig2, Lep (Leptin), Lipe, Mbtps1, Nr0b2, Nr1h2, Nr1h3, Nr1h4, Osbpl1a, Osbpl5, Ppard, Scap, Soat1, Soat2, Sorl1, Srebf1, Srebf2, Stard3, Vldlr.

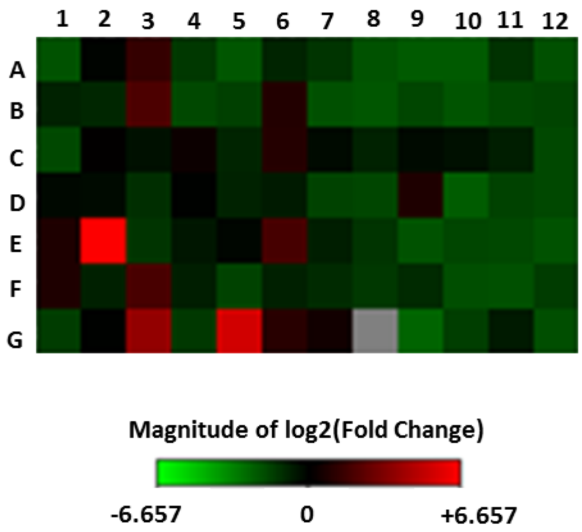

Table S2: Lipoprotein Signaling and Cholesterol Meta RT2 Profiler PCR Array list of upregulated genes. Genes in red text indicate the highly upregulated genes verified in this study. The heatmap shows the lipid pathway according to the gene list in Table S2, **with red showing increased and green showing decreased levels.** Rat neurons, rTat 50ng/ml vs con

References

Borjabad, A. et al. Significant effects of antiretroviral therapy on global gene expression in brain tissues of patients with HIV-1-associated neurocognitive disorders. *PLoS Path* **7**, e1002213 (2011).
